# Spatial and Motor Aspects in the “Action-Sentence Compatibility Effect”

**DOI:** 10.3389/fpsyg.2021.647899

**Published:** 2021-04-09

**Authors:** Alberto Greco

**Affiliations:** Department of Educational Sciences, Cognilab, Laboratory of Psychology and Cognitive Sciences, University of Genoa, Genoa, Italy

**Keywords:** action-sentence compatibility effect, ACE, embodiment, motor representation, spatial representation, action language

## Abstract

The Action-sentence Compatibility Effect (ACE) is often taken as supporting the fundamental role of the motor system in understanding sentences that describe actions. This effect would be related to an internal “simulation,” i.e., the reactivation of past perceptual and motor experiences. However, it is not easy to establish whether this simulation predominantly involves spatial imagery or motor anticipation. In the classical ACE experiments, where a real motor response is required, the direction and motor representations are mixed. In order to disentangle spatial and motor aspects involved in the ACE, we performed six experiments in different conditions, where the motor component was always reduced, asking participants to judge the sensibility of sentences by moving a mouse, thus requiring a purely spatial representation, compatible with nonmotor interpretations. In addition, our experiments had the purpose of taking into account the possible confusion of effects of practice and of compatibility (i.e., differences in reaction times simultaneously coming from block order and opposite motion conditions). Also, in contrast to the usual paradigm, we included no-transfer filler sentences in the analysis. The ACE was not found in any experiment, a result that failed to support the idea that the ACE could be related to a simulation where spatial aspects rather than motor ones prevail. Strong practice effects were always found and were carved out from results. A surprising effect was that no-transfer sentences were processed much slower than others, perhaps revealing a sort of participants’ awareness of the structure of stimuli, i.e., their finding that some of them involved motion and others did not. The relevance of these outcomes for the embodiment theory is discussed.

## Introduction

The classic cognitivist account of cognition, involving the idea that cognition can be understood as a formal manipulation of symbols, has been challenged by the embodied cognition approach, a growing area of research that reclaims the role of the body in cognitive processes, and stresses the importance of perceptual and motor aspects, and of an organism’s ability to act in its environment.

In their seminal paper, Glenberg and Kaschak (2002) found what they named the “Action-sentence Compatibility Effect” (ACE). This study provided evidence of an interaction between motor mechanisms and sentence understanding processes. In the original experiment, participants had to judge the sensibility of sentences describing actions that involved a direction toward or away from the body, and their response could be made by moving their hand in the same or in a different direction. Responses were facilitated when the hand movement required to respond was in a direction congruent with the action implied by the sentence. This effect was found with both concrete and abstract sentences.

Subsequent research that attempted to replicate the ACE had mixed success. In fact, there have been several notable failures to replicate this effect. In a wide pre-registered replication study (Kaschak et al., 2020) involving twelve laboratories, a reliable ACE could not be found. In this study the paradim adopted in all laboratories was that of Borreggine and Kaschak (2006), who explained their only partial success in their replication with the time needed in this task to prepare the motor response. The authors of the pre-registered replication concluded, however, that this result is not to be taken as conclusive because it may be related to the particular paradigm adopted. There are other known failed attempts at replication as well, although, as the authors themselves note, they were actually unpublished, with a few exceptions (e.g., Papesh, 2015; Díez-Álamo et al., 2020).

On the contrary, several studies have supported the ACE and extended its scope. Wide neurophysiological evidence has found that the motor system is involved in semantic processes (Buccino et al., 2001, 2005; Tettamanti et al., 2005). Many other studies have investigated the language-action relationship in tasks other than the original one, showing robust evidence of the action-compatibility effect. Zwaan and Taylor (2006) found it with manual rotation movements; Borghi and Scorolli (2009) with actions involving the use of hands. Aravena et al. (2010), using the event-related potential (ERP) method, found neural correlates of a compatibility effect with sentences describing actions that involved different hand positions. Awazu (2011) found the ACE in Japanese, a subject-object-verb (SOV) language, using hand and foot responses. Zwaan et al. (2012) found that the effect is related to the entire body. Asking participants to respond by leaning to the right or left side, they noticed that subjects’ trajectory was also inclined toward the rear side of the table when the sentence implied an away action and vice versa for toward sentences. Fernandino et al. (2013) studied the ACE in patients suffering from Parkinson’s disease, a disorder in the functioning of the motor system, and found that such patients were slower to understand sentences that describe an action than healthy subjects. Günther et al. (2020) showed that an effect similar to the ACE occurred also with novel concepts describing actions, which had been grounded in sensorimotor experience.

All positive results generally confirm the well-established idea that the motor system plays a fundamental role in understanding sentences that describe actions. On deeper scrutiny, however, it appears that the relevant knowledge in language comprehension does not only concern the motor component, but involves many other aspects, which cannot be easily disentangled from it: perceptual, spatial, imagery, goal-directedness, and semantic information. In order to be able to highlight the role of other factors, it seems convenient to devise tasks where the importance of the motor component is reduced. The main purpose of the present work was to set up a paradigm for exploring this issue, mainly based on the idea of using only a mouse for collecting responses in ACE tasks.

Studies presented in this paper were designed and executed over several years from 2013 in order to try to shed light on this matter. They were not published before because, overall, nonsignificant findings apparently resulted. However, as an afterthought we realized – beyond the fact that the general policy regarding replication studies has changed – that several theoretical and methodological issues can be reconsidered and take advantage of this work.

Along with the above-mentioned question of the role of the motor component with respect to other factors, some other unresolved critical questions concerning the ACE can be considered.

(1) Studies in this literature generally agree on the idea that understanding language involves the activation of perceptual and motor systems that internally “simulate” the described state of affairs. This concept of simulation, however, is not so clear because it seems to swing between a perceptual and a motor nature.
(2) There is an issue concerning the balance of locations for “yes” (the sentence makes sense) and “no” responses across presentations. Many studies did not control this balance or adopted a balance based on a within-subjects block design, i.e., reversing the location for yes-is-near and yes-is-far conditions in the middle of the experiment. Problems arise because priming and practice effects can hinder the real ACE.
(3) Typical studies in this field present sensible and nonsense sentences, along with neutral sentences as a control condition. Neutral sentences are usually discarded and not analyzed, but their analysis - compared with other kinds of sentences - can reveal interesting insights.
(4) Balancing sentences used in ACE experiments, controlling their length and difficulty, is not easy. Early experiments used methods that are now overcome by recent statistical developments, namely the use of linear mixed model techniques.

This paper is organized as follows. In the next section, the most important theoretical question will be discussed, concerning the involvement of the motor system in language comprehension, namely the concept of “simulation.” We will then examine other critical issues that in our opinion are important in ACE studies and that we can take into account in our experiments. In the ensuing sections, we will present a series of experiments basically consisting in replications of the ACE paradigm using a mouse for the response and different methods for matching mouse motion and action direction. We will consider alternative ways of analyzing data, comparing traditional methods with proposed new approaches.

## What Does “Simulation” Mean?

In the original experiment by Glenberg and Kaschak (2002), a real movement toward or away from the body was required in order to assess whether sentences were sensible^1^. As we have seen, in general, studies in the embodiment literature agree that understanding language involves the activation of perceptual and motor systems. A theoretical notion called into play in this context is “simulation” (Barsalou, 1999). According to this view, language comprehension entails internally simulating the described state of affairs. The original view posited that such simulation involves the reactivation of the same patterns of brain activation that were formed during the interaction with the environment.

Even if not all authors state it explicitly, this concept of simulation appears to be borrowed from the ideomotor principle (Greenwald, 1970; see Shin et al., 2010 for a review). According to this principle, the performance of actions is guided by perception, and actions are represented in terms of their anticipated sensory consequences, i.e., represented by their effects. Thus, associations between actions and their effects are bidirectional.

Since the ideomotor principle, a couple of similar theories have been formulated, namely the “common coding theory” (Prinz, 1997), which states that actions can also be represented in terms of their goals or effects, and the “theory of event coding” (TEC, Hommel et al., 2001), which has also been proposed as an account for the ACE. These theories posit a common representational code shared by perceived stimuli and generated actions, stating that perceiving and action planning are not distinct processes.

A similar interpretation of the ACE is the hypothesis of *motor resonance* (Zwaan and Taylor, 2006; Fischer and Zwaan, 2008). In Zwaan and Taylor’s experiments, participants were asked to make sensibility judgments of sentences expressing meanings of directionality by turning a knob. The authors found faster judgments with compatible responses, i.e., those requiring a movement in the same direction expressed by the sentence. This effect has been explained as a reactivation of past perceptual and motor experiences occurring during memory retrieval and language comprehension. This implies a sort of simulation; the term “motor resonance” has been borrowed from the mirror neuron theory, but it can result in being more ambiguous than “simulation” (Uithol et al., 2011).

However, there is a difference between the ideomotor principle, which inspires all these theories, and the ACE. The ideomotor principle points out a link between perception and planning of action, and also the classical embodied cognition approach stresses the interdependence of perception and action. In the ACE there is a further assumption, because no action or movement is perceived or observed but rather is only represented by accessing linguistic meaning.

Secora and Emmorey (2013) explored the ACE using a visual-gestural language (the American Sign Language), in order to have a gestural and then spatial input instead of purely verbal; this way the semantic aspect was separated from the spatial one. For example, in the “open the drawer” sign, participants saw the hands of the on-screen signer going away from their own body but the implied meaning was going toward. With this paradigm, these authors found that the ACE is not related to a gesture, but to its meaning, so that it is not perceptual but semantic. Then the question is: How can this semantic process enable the internal simulation to be triggered as if a real action were perceived?

The problem is that it is not always clear whether the hypothesized simulation implies a true sensorimotor-based activation for preparation or anticipation of the real motion (Boulenger et al., 2008; Dove, 2011; Jeannerod, 1994, 2001; Pulvermueller, 2005) or an abstract representation like that just related to the goal of the intended action, or to mentally imaging the motion. In other words, it is not clear whether motor mechanisms or representational aspects are prominent.

With regard to the goal of the intended action, it is worthwhile noting that, in the ACE account, some approaches lead to not distinguishing between goal-related and kinematic aspects. For example, Diefenbach et al. (2013) examined the aspect concerning the “intended goal” of action in the ACE - in their words, the “question of whether the ACE is related to the arm movement or to the intended action effect.” In this study, participants judged the sensibility of transfer sentences by producing an action that was dissociated from the intended effect because the motor response (pressing a button located toward or away from the body), for which there was no visual feedback, could produce an opposite effect on the screen (i.e., the activation of a target located in the opposite direction to that of the key pressed). According to their findings, the ACE is related to some high-level representation of the effects of action, rather than to a low-level simulation of the action itself, mainly involving motor preparation.

As to mentally imaging, some research supports the idea of a close link between imagined and actual action: For example, Papaxanthis et al. (2002), in a mental chronometry experiment, found that imagined and actual arm movements have similar durations. But these findings do not disentangle the relative contribution of representational and sensorimotor aspects. Likewise, if we consider “mentally imaging motion” as similar to what in cognitive semantics is called forming an “image schema” (Gardenfors, 2007; Lakoff, 1988), as noted by Johnson (1987), this concept also ambiguously swings between imagery and embodiment.

As we have seen, Barsalou (1999) characterizes simulation as the reactivation of “patterns of brain activation” established during interaction with the environment. But this does not necessarily involve a bodily preparation of a response, since some form of brain activation is involved in all psychological processes, including ones concerning imagery. Moreover, some neuroimaging evidence has failed to find that motor imagery and motor execution share common neural areas (Sirigu and Duhamel, 2001; Hanakawa et al., 2003; Miller et al., 2018).

Conversely, motor imagery can be connected “to other systems through which cognitive events may have an effect on performance through controlling states of arousal or by focusing attention or by priming different neuromuscular systems for action” (Annett, 1995). For example, there is empirical evidence allowing the assumption that *attentional resources* are involved in action simulation. In a different paradigm, the compatibility effect has been tested between the motion expressed in a sentence and some concurrent moving stimuli that can “match” or “mismatch” the former movement (Kaschak et al., 2005, 2006). In these cases, the ACE has not always been observed as a facilitation occurring when there is a compatibility between the two movements. These authors found a surprising *facilitation* effect in incompatible conditions (i.e., when there is a “mismatch” between sentence and concurrent stimuli) if sentence and perceptual processing overlap in time. The provided explanation was that perceptual and verbal processes in this case required access to different resources and so interference was minimized. The point here is that the simulation, supposedly grounding comprehension, here taps into attentional resources, not motor preparation.

## Critical Issues for ACE Experiments

### Disentangling Spatial and Motor Aspects

In short, the previous examination has shown that several dichotomies are at stake: goal-related versus kinematic, imagined versus actual action, perceptual (gesture) versus semantic, motor imagery versus motor execution, attention versus motor preparation. To pull the strings of all this, there are many reasons to consider the concept of “simulation,” called into play for explaining the ACE, unclear. According to O’Shea and Moran (2017), its mechanism is underspecified, and satisfactory accounts are lacking about how it is “initiated, continuously generated, and terminated, or if it is under constant conscious control” (p. 9). From what we have seen, one problem with the ACE can be summarized by the fact that whether simulation implies actual motor preparation or anticipation has been brought into question. Experiments where a full motor response is required, like the original ACE experiment and most of the subsequent ones, hardly permit this question to be disentangled. On the other hand, a simple imaginative response is not empirically verifiable. One possible solution is to require a response that is related to a spatial dimension where minimal motor effort, and then motor preparation, is required. A simple ACE task requiring a directional representation, but with a reduced motor component, can be one asking to assess sentence sensibility only by mouse movement.

The first motivation for the present work was, then, to set up experiments where a mouse is used for assessing the sensibility of sentences by moving an object (the sentence itself) on the screen, in order to see whether the ACE is still found. This should in principle be in accordance with the simulation theory, since a mouse movement does not require a full motion but can be sufficient to activate the abstract schema that is part of the simulation. Sentences expressing movement, then, should evoke the simulation of spatial relations, as long as the required motion scenario, in turn, evokes spatial relations. This can be accomplished, for example, by showing roads or corridors in perspective, by changing the word font size while the sentence is moving, etc.

We couldn’t find many attempts of this kind in literature. Lugli et al. (2012), using a setup similar to the one we are proposing, were able to replicate the ACE, but with a different paradigm, being interested in this effect with sentences referring to a social context (motion initiated by the participant and directed toward oneself or other people), but did not use sentences where the participant was the recipient of movement. A study by Papesh (2015) performed a series of experiments adopting a method very much like our own (which we had actually started independently and prior to its publication) but was unable to replicate the ACE across eight experiments and seriously challenged the ACE paradigm. Like Papesh, we wanted to replicate the ACE using mouse motion, but we also wanted to place other aspects under scrutiny, which we consider critical. We will mention these issues in the following paragraphs.

### The Confounding Effects of Practice

In Glenberg and Kaschak’s (2002) experiment, and in other typical ACE experiments as well (e.g., Glenberg et al., 2008), in order to balance the starting condition, each participant was initially randomly assigned to the yes-is-near or yes-is-far condition, and midway through the experiment, the assignment of response to a button was reversed. Even if the starting condition was balanced between subjects, in fact there were two blocks, and this left open the possibility that performance in the second block could be biased by a practice effect. This crossover could confound the ACE, because each condition (yes-is-near, yes-is-far) could be observed both in the first and the second block, thus the practice of ignoring the block order and of collapsing the two observations could lead to confounding results.

### The Problem of the Structure of Stimuli

The typical paradigm in ACE experiments envisages the use of compatible and incompatible sentences, and of neutral (filler) sentences as well. Filler items can be ones that do not involve motion, or still involve motion but are nonsense. Such neutral items are usually excluded from the analysis.

However, if response times for sensible items not involving motion were significantly different from others, this could mean that participants were somehow aware of the structure of stimuli, namely that some of them involve motion, showing “surprise” for items that do not. Thus, we set out to include filler items in the analysis as well.

In the following sections we will describe a series of experiments where we tried to replicate the ACE using a mouse for the response. The presentation of each experiment will proceed as follows. After having considered the method, we will first analyze results according to the original procedure employed in Glenberg et al. (2008). Since we found that, in general, the trimming method they used for discarding outliers leads to still having unwanted outliers and nonnormal distributions, we have in addition adopted a more restrictive procedure, further excluding response times < 300 ms and > 3000 ms after having applied the original method. Since the frequency of words and sentence length were not checked, in order to control the influence of the difficulty of sentences on reading times we analyzed all data using the linear mixed modeling (LMM; Baayen and Milin, 2010; Baayen et al., 2008) technique, which allows both participant and item variability to be accounted for. Also, since the sample size of some experiments was somewhat limited, we decided to complement the eta-squared measure of effect size with a *post hoc* simulated power using a package, based on Monte Carlo simulations, which allows the power for linear mixed models to be calculated.

## Experiment 1

This experiment was intended as a preliminary replication of the ACE, following the procedure of Glenberg et al. (2008), but requiring participants to use the mouse to move sentences away (= up in the screen) or toward themselves (= down in the screen).

### Method

#### Participants

Twenty-five students (6 male, mean age 21.6 years, sd 5.5) enrolled in introductory psychology courses at the University of Genoa took part in this experiment for course credit and were randomly assigned to group A (*n* = 13) or B (*n* = 12). All were native Italian speakers, with normal or corrected-to-normal vision. Informed consent was obtained from all participants. Participants were allowed to use their preferred hand, which was observed and recorded: all of them showed to be right-handed for mouse use.

#### Apparatus

Instructions, stimuli, response recordings, and data collection were controlled by a PC running a custom software. A 14” CRT monitor (Nek MultiSync V720 with 800x600 screen resolution) was used for displaying stimuli. Participants sat approximately 60 cm away from the display, in a separate room. Only a wireless mouse (no keyboard) was available for responses.

#### Materials

An adaptation of the stimuli of Glenberg et al. (2008), better balanced, was used. A total of 240 sentences were created, half of which were sensible and half nonsensical. Both sets of sentences were further divided into concrete and abstract. All sentences were in the simple present tense to maximize the simulation of actual action, as suggested by Glenberg et al. (2008). The sentence distribution is shown in Table 1, and the full list of sentences is in Supplementary Appendix.

**TABLE 1:** Distribution of sentences in ACE experiments.

**Sensibl.**	**Abstr.**	**Direction**	**#of sentences**
Nonsense			120
	Abstract		60
		Away	20
		No transfer	20
		Toward	20
	Concrete		60
		Away	20
		No transfer	20
		Toward	20
Sensible			120
	Abstract		60
		Away	20
		No transfer	20
		Toward	20
	Concrete		60
		Away	20
		No transfer	20
		Toward	20
Total			240

#### Procedure

**FIGURE 1:**
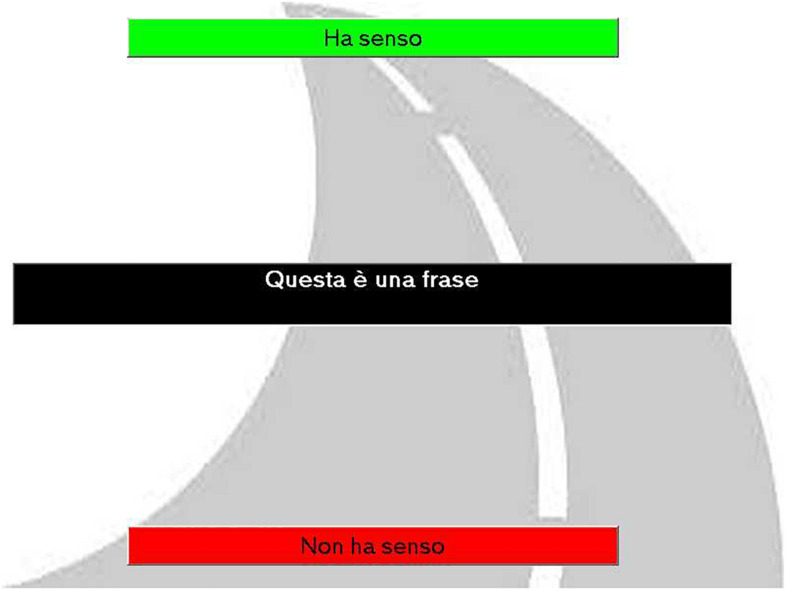
Screenshot of exp. 1 background.

A picture of a road that led away from the subject was used as the screen background for this task, to increase the impression of distancing (Figure 1). Each sentence appeared at the center of the screen, in white characters inside a black rectangle. Words were written in MS Sans Serif font 18 points, in bold. Two boxes also appeared at the same time, at the top and bottom of the screen, respectively; one was green and labeled “HA SENSO”(It makes sense), the other was red and labeled “NON HA SENSO” (It does not make sense).

Participants were randomly assigned to one of two subgroups (A and B), differing only as regards the locations (far-near) assigned for indicating sensible and nonsensical responses at the beginning of the experiment, which were opposite in the two subgroups. Midway through the experiment, participants were instructed to reverse the assignment of response locations and were given additional practice.

Participants first practiced with the system by dragging 10 times one of the sentences “Ha senso” or “Non ha senso,” shown randomly, and dropping it into the corresponding boxes. After assurance that the operation had been well understood and executed, the main task started, where experimental sentences were presented in a random order that was different for each participant.

For Group A, in the first block of 120 sentences, the yes-is-far (YF) response (green box) was set to the away (upper screen) direction and the yes-is-near (YN) response (red box) was set to the toward (bottom screen) direction. In the second block of 120 sentences the direction was inverted (YF set to the bottom screen and YN to the upper screen). Conditions were fully inverted for Group B (1st block YF = bottom, YN = upper; 2nd block YF = upper, YN = bottom).

A black rectangle was shown in the center screen and one sentence appeared when clicked. Participants then judged whether the current sentence was sensible or nonsensical, by dragging the rectangle and dropping it into the corresponding box. Times were recorded from the sentence appearance to dragging start (*judgment times*, later simply referred as “reaction times,” RTs), and also from then to the drop on the final location. When dropped, the current sentence disappeared and a new black box was shown. The latter measure was not used in analyses, both for theoretical and practical reasons. On the theoretical side, the ACE assumption entails simulation being already elicited during sentence *reading*, and even has a role in sentence understanding. On the practical side, times required to drop sentences would be affected by spurious factors, e.g., like mouse deviations from the shortest trajectory, and thus analysis of such times would not make much sense.

### Results and Discussion

#### Standard Analysis

Data were first cleaned according to Glenberg et al.’s (2008) Exp 1 standard procedure. In detail:

(a) the number of errors in sensible sentences was checked, in order to exclude participants who committed more than 10% of errors (no subject was excluded in our experiment);
(b) the first 12 items in each block were eliminated;
(c) the incorrect responses were eliminated;
(d) for each participant, in each of the six conditions defined by the two abstractness levels and three sentence direction levels, means and standard deviations were computed, and judgment times greater than 2.5 standard deviations were eliminated.

The main aim of our analysis was to look for a significant ACE. Judgment times, as previously defined as times from when the box was clicked and the sentence appeared to when the box movement started, were recorded as response times (RTs). RTs thus measured the time required for the evaluation processing (understanding the sentence meaning, judging its sensibility, and deciding on the response). Glenberg et al. (2008) found shorter RTs for toward sentences when the required response was toward the participant, but not with the away sentences/yes-is-far combination.

This means that they found the ACE only with toward sentences, but they did not explain why the effect did not hold with the other compatible condition. Actually, a true ACE should consist in the difference between sensible toward (T) and away (A) sentences, *both* in YN and YF response conditions. For this reason, in our analyses we considered all conditions where the directions implied in the sentence and in the response matched, labeling them as “compatible,” and where they did not match as “incompatible.”

Given the results of this first experiment, we did not perform any statistical analysis on these data from the compatibility point of view, because already from simple inspection the outcome was very different than expected, and in fact absolutely no difference was found between compatible (mean RT 1502.73 ms) and incompatible (1505.42 ms) conditions.

#### Extended Analysis

However, we wanted to explore in more detail the effect of single factors. Since the trimming procedure adopted by Glenberg et al. (2008) is not able to cut out unwanted outliers (the resulting extreme values were min. 93 ms, max, 5329 ms; also, the distribution was far from normal, having a kurtosis value of 4.35), we decided to further trim data considering RTs < 300 ms and RTs > 3000 ms as outliers. This way, the resulting distribution had minimal skewness (0.96) and excess kurtosis (0.62) values (well within the limits recommended by Griffin and Steinbrecher, 2013).

The frequency of words included in sentences was not controlled in this experiment, and neither was sentence length. This obviously leaves open the possibility that the difference in the difficulty of sentences may have influenced the reading times. This is a common problem in tasks including linguistic stimuli (Brysbaert, 2007). The linear mixed modeling (LMM; Baayen et al., 2008; Baayen and Milin, 2010) technique, however, is able to overcome this problem by allowing both participant and item variability to be accounted for.

We used the *lme4* package in the R 3.3.3 environment^2^, and for obtaining F-statistics we used the ANOVA function with the *lmerTest* package^3^. Degrees of freedom for reported F-values were estimated with a Satterthwaite approximation. Eta-squared values were computed using the function *eta_sq* of the *sj_stats* package.

We built a model with RTs as dependent variable, and participants and items as random factors. Fixed factors were Group as between-subjects factor, and Block, Type (Abstract, Concrete), Sentence Direction (Away, Toward), Response Direction (Yes-is-Near, Yes-is-Far), Sentence Direction X Response Direction interaction as within-subjects factors (see Supplementary Appendix Table 1a, for R scripts and details about the procedure).

Main effects were found for Block (Means: Block 1 = 1507.41, Block 2 = 1352.96; *F* = 34.89, SE = 27.41, df = 76.55, *p* < 0.001, η^2^_*p*_ = 0.31) and for Sentence Direction (Means: Away = 1396.70, Toward = 1465.53; *F* = 6.79, SE = 32.69, df = 153.05, *p* < 0.05, η^2^_*p*_ = 0.08). Given this important effect of practice, we repeated the same analysis separately by block, with the same result for Block 1 (sentence direction was the only significant factor as for the general analysis), and no significant factor resulting for Block 2 (details in the Supplementary Appendix Tables 1c,d).

We also built an identical model including no-transfer sentences - sensible but expressing no direction (e.g., “You look at the box with Laura” or “You and Peter read a book”). Results were very similar to previous ones, with main effects for Block and Sentence Direction, but even more marked (see Supplementary Appendix Table 1d). Particularly remarkable is the fact that much greater RTs resulted for no-transfer sentences (Mean: 1495.96; *F* = 8.36, SE = 33.9, df = 230.38, *p* < 0.001, η^2^_*p*_ = 0.13). Note that, while in Glenberg’s original experiment (Glenberg and Kaschak, 2002) there were no sentences without direction, Glenberg et al. (2008) found higher times for the “no transfer” sentences but they did not explain why. We come back to this later.

Coming to the main results, the big facilitation for the second block is easily explained by the effect of practice. The absence of interaction between Sentence and Response directions confirms that the ACE is not revealed by this experiment, but longer RTs resulting for YN sentences can explain why. In fact, this is a surprising effect and might have been due to the construction used in the toward sentences, where the construal “a te” was used instead of the more common “ti”: for example, “Anna dà la palla a te” (Anna gives the ball to you) instead of “Anna ti dà la palla” (Anna gives you the ball). This construal might have been more difficult to process. In order to overcome this difficulty, we planned a second experiment where we modified the expression “a te” with the more natural expression “ti.”

Before moving on the next experiment, we want to hint at the possibility that this first experiment was underpowered. In fact, we considered this experiment to be a pilot experiment and ran a *post hoc* simulated power using the *simr* R package (Green and MacLeod, 2016), which allows the power for linear mixed models from the *lme4* package to be calculated, based on Monte Carlo simulations (see Supplementary Appendix Table 1f). The resulting power curve for Block after 1000 simulations revealed that a sample of 15 participants would have already been sufficient for reaching a power of 98.5% (confidence interval 97.5–99.2%), and for Sentence Direction the smallest sample size required to satisfy the 80% power level was close to 25 participants (power of 75%, confidence interval 72–80%).

## Experiment 2

### Changes to the Previous Experiment

This experiment was planned in order to make up for the difficulty arising from the construal of some sentences. As explained above, the grammatical construal of toward (YN) sentences was changed, using the “ti” (you) construction instead of “a te” (to you). An additional setup of sentences was also made, with removal and replacement of uncommon or ambiguous ones; also, the regular matching of person names with sensible/not sensible sentences was eliminated by changing some person names.

### Method

#### Participants and Procedure

Twenty-four students (1 male, mean age 20.8 years, sd 1.3, all right-handed for mouse use) participated for course credit and were randomly assigned to group A (*n* = 12) or B (*n* = 12). For all participants the same requirements as for Exp 1 were met. The procedure was identical to that of Exp 1.

### Results

#### Standard Analysis

As in the previous experiment, we applied the standard cleaning procedure adopted by Glenberg et al. (2008), and we labeled as “compatible” all conditions where the directions implied in the sentence and in the response matched, and where they did not match as “incompatible.” No participant committed more than 10% of errors in sensible sentences.

By adopting this analysis, we could not find any difference in this experiment either between RTs in compatible (mean RT = 1411.55 ms) and incompatible (mean RT = 1416.38 ms) conditions.

#### Extended Analysis

We thus proceeded to explore the effect of single factors. We again trimmed data in a more restrictive manner considering, in already cleaned data, RTs < 300 ms and > 3000 ms as further outliers (the resulting distribution skewness was 0.99, and excess kurtosis 0.94). We built a model identical to that of Exp 1, with the same fixed and random factors (see Supplementary Appendix Table 2a). Block was the only main effect (Means: Block 1 = 1473.41, Block 2 = 1318.31; *F* = 22.07, SE = 32.22, df = 72.87, *p* < 0.001, η^2^_*p*_ = 0.23). This result again shows an evident facilitation effect due to practice, but no ACE. Given the result of the previous experiment (and of Glenberg et al., 2008) about no-transfer sentences, we inquired whether this was the case too in this experiment, so we repeated the analysis including no-transfer sentences. In this case, too, an effect for Sentence Direction emerged, due to a greater judgment time for no-transfer sentences (Means: Away 1386.62, Toward 1402.08, No-transfer 1512.66; *F* = 10.45, SE = 38.2, df = 186.22, *p* < 0.001, η^2^_*p*_ = 0.16; Supplementary Appendix Table 2b). Given the effect of practice, we again made a separate analysis by block. A significant facilitation was found for the yes-is-far direction in the second block, possibly due to specific conditions pertaining to this particular task execution, but no ACE resulted for either single block (details in the Supplementary Appendix Tables 2c,d).

Once again, we wanted to explore the possibility that this experiment was underpowered, running a power simulation using the *simr* R package; again a sample of 15 participants would have already been sufficient for reaching a high power level (96.9% for Block, 96.7% for Sentence Direction; Supplementary Appendix Table 2e).

## Experiment 3

### Changes in This Experiment

**FIGURE 2:**
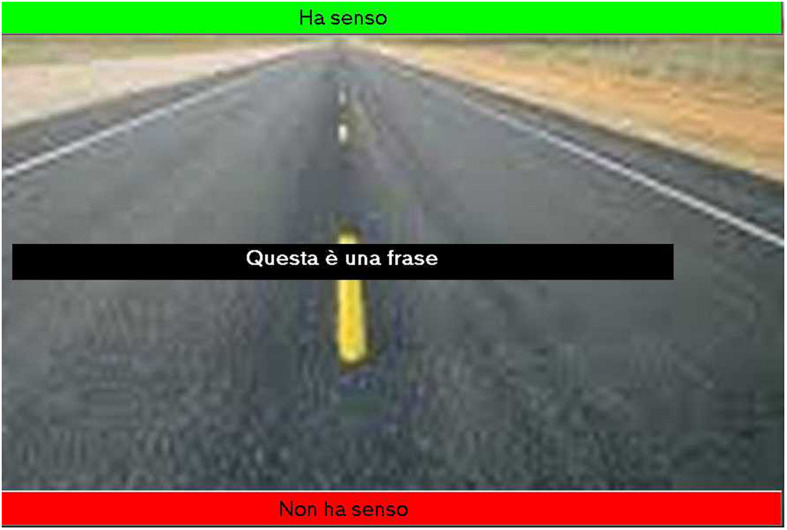
Screenshot of exp. 3 background.

In order to explain the failure to reach the ACE, we hypothesized that the context was not so clearly suggesting a sense of toward or away and decided to use a more perspicuous background. The screen background was changed to increase the sense of depth (Figure 2).

### Method

#### Participants and Procedure

Twenty students (7 male, mean age 22.7 years, sd 5.7, all right-handed) participated for course credit and were randomly assigned to group A (*n* = 10) or B (*n* = 10). The procedure was identical to that of previous experiments.

### Results

Data were analyzed in the same way as before, according to Glenberg et al.’s (2008) Exp 1 procedure. No participant committed more than 10% of errors in sensible sentences.

Exactly as in previous experiments, no difference was found between compatible (Mean RT: 1468.17 ms) and incompatible (Mean RT: 1464.36 ms) conditions.

We thus proceeded with extended analysis, considering RTs < 300 ms and RTs > 3000 ms as further outliers (resulting distribution skewness = 0.92; excess kurtosis 0.74). The data were then submitted to mixed linear model analysis following the same design as in previous experiments (Supplementary Appendix Table 3a). Also, in this case, only the effect of Block resulted in being significant (Means: Block 1 = 1509.64, Block 2 = 1359.57; *F* = 21.84, SE = 31.86, df = 72.41, *p* < 0.001, η^2^_*p*_ = 0.23), showing once again no ACE and a facilitation occurring in the second block due to practice. Even in this case, a separate analysis by block did not reveal any ACE (details in the Supplementary Appendix Tables 3b,c).

As before, we tested whether increased RTs occurred with no-transfer sentences (Supplementary Appendix Table 3d). In fact, this was again the case (Means: Away 1435.45, Toward 1432.51, No-transfer 1614.27; *F* = 21.29, SE = 39.26, df = 194.22, *p* < 0.001, η^2^_*p*_ = 0.28). When including no-transfer sentences an effect of Type was found, due to a facilitation for concrete sentences (Means: Abstract 1518.33, Concrete 1467.12, *F* = 4.43, SE = 27.62, df = 107.79, *p* < 0.05, η^2^_*p*_ = 0.04). Given that this effect came only after including no-transfer sentences, we tested whether an interaction was present between the direction of sentences and concreteness, but this was not the case. Power simulation showed that a sample of 15 participants would have been sufficient for reaching a power level of 99.1% for Block, the full sample for a power level of 80% for Sentence Direction; the simulation did not show a reliable power level for the predictor Type – details in Supplementary Appendix Table 3e).

The failure to obtain any ACE in this experiment either led us to suppose that the problem was the lack of an adequate automatization that would bring the subjects to associate the upward direction on the monitor with the “away” sense and the downward direction with the “toward” sense. We then decided to try to make the ACE task be preceded by a warmup task, which consisted in the exercise of moving a box with the name of an object in the away or toward directions.

## Experiment 4

### Changes in This Experiment

As explained above, in this experiment, before undergoing the main task, participants were requested to perform a warmup task first, with the aim of getting them acquainted with the association of the upward direction on the screen with “away from themselves” and of the downward direction with “toward themselves.”

**FIGURE 3:**
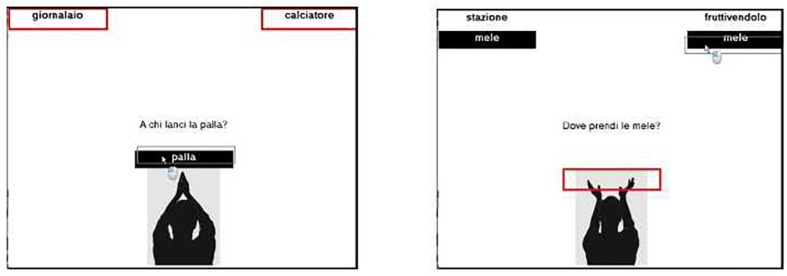
Screenshots of exp. 4 warmup task. **Left:** [inside red rectangles] newsagent, footballer; [question in the center] To whom do you throw the ball? [inside black box] ball. **Right:** [top words] station, grocer; [inside black boxes] apples; [question in the center] Where do you get apples? (the mouse icon was part of the animation in the demonstration clip and was not shown in the task).

For the away direction, the name of an object, written in white font inside a black box, appeared in the center, immediately above the silhouette of a person depicted in the act of throwing something. The participants had to move the black box to one of the two red boxes at the top of the screen, according to the sense of a simple question. In the example (see Figure 3, left) the question was “To whom do you throw the ball?” and the word “palla” (“ball”), written in the black box, had to be moved up to one of the two option boxes (“newsagent” or “footballer”) at the top of the screen. For the toward direction, two words appeared on the two sides at the top of the screen; below them, two black boxes with the name of an object, written in white font, had to be moved down to a red box placed immediately above the silhouette of a person depicted in the act of taking something. A video clip was shown with instructions, which illustrated with animations the execution of the task. Twenty-six items (half toward, half away) were then presented in random order.

**FIGURE 4:**
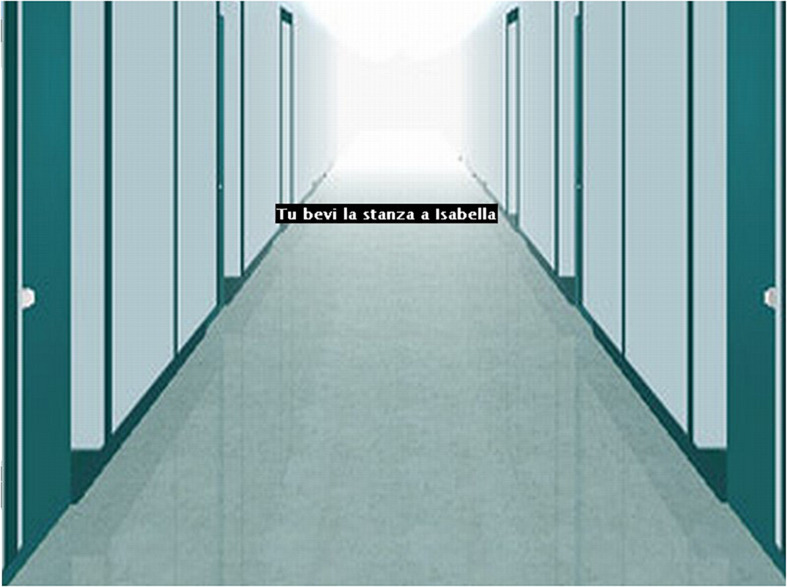
Screenshot of exp. 4 background.

As to the main ACE task, the background was changed to an even more perspicuous one, depicting a corridor in perspective (Figure 4). Moreover, to increase the impression of movement, the size of words was scaled down and up while the black box was moved in either direction and, to increase the sense of effort related to the movement, the mouse speed was significantly slowed.

### Method

#### Participants and Procedure

Twenty students (8 male, mean age 24.6 years, sd 7.4, all right-handed) participated for course credit and were randomly assigned to group A (*n* = 10) or B (*n* = 10). The sentences and procedure were identical to those of previous experiments.

### Results

The data analysis procedure remained the same as in previous experiments. No participant committed more than 10% of errors in sensible sentences. Since, as mentioned, mouse speed was reduced significantly to increase drag effort, from this experiment onward all response times became longer. As in previous experiments, if the standard Glenberg et al. procedure was adopted, no difference could be found between RTs in compatible (mean RT = 1762.53 ms) and incompatible (mean RT = 1755.51 ms) conditions. After having further trimmed data, we first checked the shape of the resulting distribution, which was not too dissimilar to normal (skewness 0.74, excess kurtosis 0.01). We then built a mixed linear model similar to ones used previously, and obtained very similar results (Supplementary Appendix Table 4a), with Block being the only significant factor (Means: Block 1 = 1736.54, Block 2 = 1563.57; *F* = 36.56, SE = 29.49, df = 72.32, *p* < 0.001, η^2^_*p*_ = 0.34). No ACE could be found again, only the effect of practice in the second block, as always. In a separate analysis by block, significantly faster RTs resulted in the first block for yes-is-near sentences and in the second block for yes-is-far sentences, irrespective of any other factor. This is probably a local effect, because no interaction with sentence direction was found, showing again no ACE (details in Supplementary Appendix Tables 4b,c). Power simulation showed that for the Block result, 11 participants would have been sufficient for reaching an 88% power level (Supplementary Appendix Table 4d).

Also, in this experiment, longer RTs occurred with no-transfer sentences (Supplementary Appendix Table 4e; Means: Away 1624.99, Toward 1671.55, No-transfer 1799.67; *F* = 17.90, SE = 39.84, df = 238.95, *p* < 0.001, η^2^_*p*_ = 0.25). Power simulation showed that for this result too a power level of 99% would have been achieved with just 11 participants.

The effect of Type was significant when including no-transfer sentences, showing that concrete sentences were easier (Means: Abstract 1723.79, Concrete 1667.39, *F* = 5.76, SE = 26.36, df = 105.1, *p* < 0.05, η^2^_*p*_ = 0.05), but no interaction resulted between Type and Sentence Direction (Supplementary Appendix Table 4e).

The results of this experiment confirm that the ACE continues not to be found using a mouse for moving sentences, even after having introduced a warmup task intended to strengthen the association of upward direction with self-distancing and vice versa for downward direction. We then explored the possibility that with the vertical screen orientation this association was difficult anyway, and planned a new experiment to be performed with the monitor placed horizontally.

## Experiment 5

### Changes in This Experiment

The aim of this experiment was to see whether the ACE could be obtained by rotating the monitor and placing it horizontally on the table.

### Method

#### Participants and Procedure

Twenty-eight students (5 male, mean age 22.8 years, sd 6.8, one left-handed) participated for course credit and were randomly assigned to group A (*n* = 15) or B (*n* = 13).

The procedure was the same as in Experiment 4’s main task (no warmup task was performed), including mouse slowing and font size scaling, except that in this experiment the monitor was rotated and placed flat on the table. A 17” LCD monitor (LG Flatron T1708 with 800x600 screen resolution) was used for displaying stimuli. This device was not used as a touchscreen monitor, but – as in previous experiments – sentences could be moved using a mouse placed alongside on the table.

### Results

The same data analysis procedure as in previous experiments was performed. No participant committed more than 10% of errors in sensible sentences. Also, in this experiment, again, no difference was found between RTs in compatible (mean RT = 1601.16 ms) and incompatible (mean RT = 1613.49 ms) conditions. With further data trimming the resulting distribution was almost normal (skewness 0.59, excess kurtosis −0.28). The mixed linear model, as in all other experiments, did not show any ACE since there was no interaction between sentence and response directions (Supplementary Appendix Table 5a). Block was again a significant factor (Means: Block 1 = 1721.92, Block 2 = 1603.50; *F* = 13.14, SE = 33.64, df = 74.08, *p* < 0.001, η^2^_*p*_ = 0.15). Response direction resulted in being a significant factor in this experiment, as yes-is-near sentences turned out to be more difficult (Means: Yes-is-Far = 1619.45, Yes-is-Near = 1702.05; *F* = 15.52, SE = 29.94, df = 1544.22, *p* < 0.001, η^2^_*p*_ = 0.01). Separate analysis by block confirmed this result (Supplementary Appendix Tables 5b,c). The reason for this effect remains to be explained. We hypothesized that it was due to a local condition, because the space on the table for moving the mouse toward the subject may have turned out to be too limited. More importantly, it is noteworthy that, if no-transfer sentences are included in data analysis, RTs for this kind of item resulted in being longer once more (Means: Away 1656.25, Toward 1666.93, No-transfer 1813.6; *F* = 15.84, SE = 40.83, df = 232.38, *p* < 0.001, η^2^_*p*_ = 0.22; Supplementary Appendix Table 5d).

## Experiment 6

### Method

#### Participants

Forty-two students (7 male, mean age 21.1 years, sd 1.8, 2 left-handed but right-handed for mouse use) participated for course credit and were randomly assigned to group A (*n* = 21) or B (*n* = 21).

#### Procedure

In this experiment, beyond dealing with the issue concerning the mouse motion on the table in Experiment 5, we also wanted to make the experiment even more similar to that of Glenberg et al. (2008). Thus, the procedure was like in Exp 5, with the monitor rotated and placed on the table, but in this experiment instructions were changed to encourage participants to consider sentences as concerning their person: Sentences that appeared as examples in practice trials were referred to the person (e.g., “this is *your* first sentence,” “*your* sentence is sensible,” etc., italics not shown to participant) and, after explaining – as in all experiments – that the participant should judge if the sentence was sensible or not, the sentence “In any case, consider it as referring to your person” was added. Furthermore, participants were explicitly instructed to respond quickly.

### Results

Data were analyzed in the same way as in previous experiments (Supplementary Appendix Tables 6a–f). With the classical method, no significant difference resulted in RTs for compatible and incompatible conditions (Means: Compatible 1795.04, Incompatible 1802.44; *F* = 0.03, df 2700, *p* = 0.86)^4^.

With our more strict trimming method, the distribution approached normality (skewness 0.72, excess kurtosis 0.12). The mixed linear model once again did not show any ACE. Block was significant as always (Means: Block 1 = 1781.84, Block 2 = 1605.04; *F* = 43.01, SE = 27.02, df = 71.27, *p* < 0.001, η^2^_*p*_ = 0.38) and in this case no significant effects resulted from separate analysis by blocks. Importantly, as in all experiments, when including no-transfer sentences, these items resulted in being more difficult (Means: Away 1679.38, Toward 1704.74, No-transfer 1816.45; *F* = 13.91, SE = 34.49, df = 177.57, *p* < 0.001, η^2^_*p*_ = 0.21). Also, RTs to abstract sentences and yes-is-near sentences were significantly slower, but the eta-squared value was too small to consider the effect size for this result satisfactory.

## Final Analysis

**FIGURE 5:**
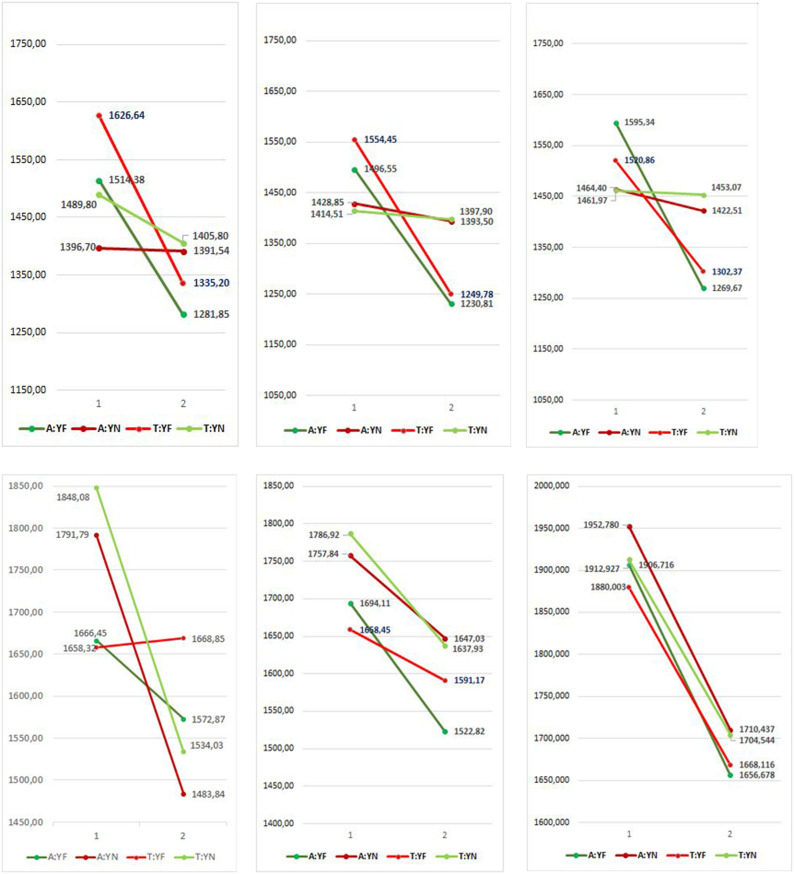
Sentence direction (A,T) and Response direction (YF,YN) interaction in all experiments (mean RTs). 1 and 2 indicate Block. Interactions denoting ACE are in green, negative ACE in red.

Statistical analyses performed did not show a significant ACE in any of the experiments. Figure 5 summarizes graphically all results, showing RTs of Sentence direction and Response direction in the two blocks for all experiments.

Our experiments had mostly an exploratory nature, and therefore each one was characterized by a somewhat reduced sample size. However, the general structure of all experiments remained almost the same. The same sentences were always used (except in the first experiment), and the treatment by blocks and groups remained the same; changes were only aimed at ensuring that direction of motion was better associated with the required mouse movement. Thus, as a final step we tried to make a pooled analysis of all experiments (excluding the first because of the diversity of sentences).

The analysis was the same as that made for each experiment, using the extended cleaning procedure, considering all sentences (including no-transfer ones), but including the experiment version as a random factor (see Supplementary Appendix Table 9). Pooled sample size was 134. Given the form of distribution, no log transformation was deemed necessary. Results substantiated very closely previous outcomes. No interaction was found between sentence direction and response direction (*F* = 0.58, *p* = 0.56). Highly significant effects were found again for block (Means: Block 1 = 1701.19, Block 2 = 1551.71; *F* = 116.29, SE = 14.87, df = 228.69, *p* < 0.001, η^2^_*p*_ = 0.34) and sentence direction (Means: Away 1579.23, Toward 1614.28, No-transfer 1685.42; *F* = 21.63, SE = 19.3, df = 283.74, *p* < 0.001, η^2^_*p*_ = 0.16). Our results do not confirm the so-called “looming effect” (Díez-Álamo et al., 2020), i.e., the facilitation found for sentences expressing motion toward themselves. This might be due to the different sentences employed, but as it is a newly found effect it deserves additional investigation. A further result was a type effect, showing some advantage for concrete sentences (Means: Abstract 1642.72, Concrete 1607.95; *F* = 7.62, SE = 14.76, df = 237.09, *p* < 0.01). This is consistent with what has already been found (Glenberg and Kaschak, 2002; Glenberg et al., 2008; Díez-Álamo et al., 2020), but it should be noted that the effect size was small (η^2^_*p*_ = 0.03).

To make sure that some differences in the construction of neutral and no transfer sentences used from experiment 2 onward (those of the first were different) were not a confusing factor, although the length of the sentences was substantially identical between the two types of sentences (the length of *all* sentences was 28 or 29 characters) we checked also the readability index of the sentences using the formula of Flesh adapted to the Italian language (Franchina and Vacca, 1986)^5^.

Finally, in order to to better evaluate the strength of the evidence for the null hypothesis (H0) over the alternative hypothesis (H1) about the interaction effect between Sentence direction and Response direction, we conducted a Bayesian analysis on each experiment, except the first one. Bayes factors provided strong evidence in favor of the null models (BF_01_ for the interaction was always very high). The results and details are shown in Supplementary Appendix Table 9.

## General Discussion

This study was stimulated by some problems of the standard ACE paradigm, namely the difficulty of disentangling spatial and motor aspects in the assumed underlying simulation process, the fact that results of experiments spanning in two blocks may be confounded by the practice effect, and the puzzling disadvantage in processing no-transfer sentences in the context of ones expressing transfer. The first motivation for this suite of experiments was then to explore whether the ACE could be found when a mouse was used for moving sentences expressing motion.

It may be asked to what extent using a mouse as the response mode reduces the motor component of the ACE. In general, there are two possible limb positions in the use of a mouse: a distal position, with the forearm rested on the desk, and a proximal position, with only the wrist rested on the desk (Sako et al., 2017). In all our experiments the mouse was placed far enough away from the edge of the table, substantially favoring a distal position. Therefore, the use of mouse required participants to rest their forearm on the table, maintain grasp but without transport (Jeannerod, 1984), and abduct and slightly rotate their arms to drag the object on the screen. In any case, even though the participants might have raised the arm slightly to move the mouse, arm extension would still be smaller than that required by the displacement of the entire arm, as required to press a button on a keyboard (besides, placed on their lap in the original experiment). A comparison regarding the effort required by using mouse and keyboard (Bruno-Garza et al., 2012) showed that when compared to mouse activity, there was a 50% increase in the right trapezius muscle effort with keyboard use, which was also associated with increased wrist velocity and acceleration values, and wrist ulnar deviation; moreover, the shoulder rotation change was 25 degrees with keyboard use and 15 degrees with mouse. Thus, the motor effort appears clearly greater with keyboarding than with mousing.

That being said, in none of our experiments we were able to find the ACE using a mouse, with either the original paradigm (Glenberg and Kaschak, 2002; Glenberg et al., 2008) or with the modifications introduced. We supposed that the result of the first experiment was biased by a particular grammar construction of indirect pronouns in Italian (e.g., “give to you”) instead of direct (“give you”), but this turned out to be not the case after having changed this construction (Exp. 2). Thus we changed the background to give a greater impression of depth (Exp. 3) but no ACE was observed. We then supposed that asking participants to move sentences along an upright monitor might not encourage them to perceive the vertical dimension as an approaching or distancing one. We therefore (Exp. 4) tried to better establish this association by performing a warmup task, asking participants to repeatedly and explicitly move items away or toward themselves according to simple questions. Furthermore, in order to enhance the impression of depth, other expedients were used. The size of words was increased when moved toward or decreased when moved away; the mouse speed was slowed down; the background was changed again. Notwithstanding all these changes, the compatibility effect continued not to occur. And the same thing also happened even when the monitor was placed horizontally on the table (Exp. 5) and when changes were made to the sentences in order to make them more personally relevant to the participant (Exp. 6).

On the other hand, the most relevant result, in all six experiments, was always a strong difference between blocks. The most plausible explanation is that this effect was due to the practice, and in any case this shows how confounding the practice of ignoring the block order may be. Fallacious results may be obtained, especially when a mixed within- and between-subjects design is adopted, so that the starting condition of the response direction (YN and YF) is manipulated between subjects and reversed midway within subjects. In this case, where each condition of response direction is observed in both blocks, the effect of the easier condition will be counterbalanced by the greater difficulty in the first block, and the effect of the most difficult condition by the greater ease in the second block. Thus, collapsing the two observations makes real the risk that they cancel each other out.

Furthermore, there are other reasons why practice effects should not be neglected. It is possible that responses required in ACE experiments, due to their repetitive nature, can be treated like action sequences. The Dual Processor Model (DPM) of sequence production developed in the action sequences literature (Abrahamse et al., 2013; Ruitenberg et al., 2015) can explain why shorter times are invariably observed in the second block of typical ACE experiments.

According to the DPM, sequencing performance involves sequence retrieval and motor buffer loading by a cognitive processor (i.e., preparation processes), followed by the fast execution of the motor buffer content by a dedicated motor processor (i.e., execution processes). The precise content that is loaded into the motor buffer changes with practice. Initially, the cognitive processor loads each individual element – that is, key press – by translating each stimulus into the appropriate response, which is then directly executed by the motor processor. With practice, motor chunks develop, i.e., representations of a series of successive responses that can be retrieved and loaded as if they were a single response. The cognitive processor can thus select and load such a chunk into the motor buffer as a whole, after which the motor processor executes all elements within the chunk. This means that the response time on the first key press of a motor chunk reflects selection, retrieval, and execution (i.e., preparation phase), while response times on later key presses primarily reflect execution processes (i.e., execution phase) because motor chunk selection and retrieval have already occurred (Ruitenberg et al., 2015).

However, even in the absence of an ACE, a different, more subtle sort of embodiment effect might have emerged from all our experiments. In fact, a constant result found in all experiments was a strong slowdown in performance with no-transfer sensible sentences. This finding seems to show that the common practice of excluding such sentences from analysis could be misguided. This might be evidence that, after all, the participants were aware of the structure of stimuli, namely that some of them involved motion and others did not. Thus, judgment times for sensibility were longer when sentences did not involve any direction, placed in the context of sentences that did imply one, and this may be considered a weak embodiment effect, considering that a motor response – albeit weak – was required in any case. Longer RTs for no-transfer sentences, perhaps, could be due to a sort of “surprise” that impacted processing resources. It’s worth noting that, while in Glenberg’s original experiment (Glenberg and Kaschak, 2002) there were no sentences without direction, Glenberg et al. (2008) also found higher times for “no-transfer” sentences but, as we have noted previously, they did not explain why.

In order to analyze this finding more deeply, a comment is needed on what kinds of sentences should be considered in results. Among the stimuli presented in our experiments, there were two kinds of “filler” sentences, namely “nonsense” and “no-transfer” ones. Having found, as just mentioned, higher RTs for no-transfer, but sensible, sentences, we reasoned that it would be worth analyzing also the other kind of “filler” sentences that are usually excluded from analyses, according to typical ACE paradigms, namely sentences without meaning but expressing a direction (like “Anna throws the theory at you”). Generally speaking, it would be likely that nonsense sentences would be processed worse, but no effect of meaningfulness on RTs was found in our experiments (see Supplementary Appendix Tables 7, 8). In fact, in all experiments there was no overall difference between sensible and nonsense sentences. Rather, there was a constant interaction of meaningfulness with sentence direction, since no-transfer sentences were processed faster when they were nonsense than when sensible. The same pattern resulted in all experiments, and always reached statistical significance from the third experiment forward. This seems counterintuitive, but it is easily explained by the fact that nonsense sentences could be quickly dismissed and thus required less processing, while the lack of direction was detected more straightforwardly in sensible sentences. This unfulfilled expectation combined with the awareness of the contrast between “transfer” and “no-transfer” sentences may have been the cause of the highest RTs with these kinds of sentences in all experiments. Of course, it remains questionable whether this effect may be interpreted as a genuine embodiment effect or if there may be some other explanation. For example, a similar effect was found in a different paradigm, aimed at verifying the modality-switch effect on perceptual simulation (Scerrati et al., 2015). In this study, a facilitation was found when a response to a target sentence was in the same modality (aural or visual) elicited by a previous prime sentence, but hindrance occurred when primes were not perceptually informative. The authors explained this finding suggesting that neutral primes did not provide the benefit of triggering a perceptual simulation, resulting in slower response times.

## Conclusion

In conclusion, putting together all our results, the ACE was never found in six experiments where the mouse was used for assessing the sensibility of sentences. Since this mode of response requires minimal motor effort but still a full spatial representation, our study overall failed to support the idea that the ACE could be related to a simulation where *spatial* aspects rather than *motor* ones prevail. This would imply that simulation of spatial relations did not occur in our experiments, or if it occurred it was not strong enough to show a compatibility effect. Only a possible simulation of this kind might be hypothesized as an explanation for the anomalous RTs found with no-transfer sentences, but perhaps further, more detailed investigation would be needed in order to be able to establish this interpretation with confidence.

These results do not necessarily support, however, the idea that motor aspects of the ACE prevail over spatial ones when the ACE is found. It depends, of course, on how strong the evidence about the ACE is considered to be. In fact, as is known, there is a lively ongoing debate about conclusive evidence in the literature of a robust ACE (Mahon and Caramazza, 2008; Postle et al., 2008; Chatterjee, 2010; see Meteyard et al., 2012 for a review; Papesh, 2015; Goldinger et al., 2016; Mahon and Hickok, 2016). Some support for the importance of motor simulation, however, seems to be provided by the results of Moretti and Greco’s (2018) experiments. In this study, participants, when evaluating the truth value of sentences, in one condition moved sentences on a computer screen by performing the head gestures of nodding and shaking, while participants in another condition were required to move sentences with the mouse instead of with the head. Results showed a compatibility effect with head movements only. In fact, reading times were shorter when sentences expressing truth were moved by nodding and longer with shaking (and vice versa with false sentences). Such a compatibility effect was not found with mouse motion, suggesting that simulation, if it occurred, was motor and not spatial. The interest of this kind of experiment as a countercheck also lies in the fact that a motor response in a spatial environment was required but there was no action-related meaning.

The ACE paradigm has met with several confirmations and disconfirmations, but each time under different conditions and with different aspects in mind. An embodiment research program should better dissect the relationships and points of contact between different paradigms. Perhaps it would be necessary in the future to better identify what constraints cause the effect to be found or not. In any case, it is research that is still worth doing.

## Data Availability Statement

The original contributions presented in the study are included in the article/Supplementary Material, further inquiries can be directed to the corresponding author/s.

## Ethics Statement

Ethical review and approval was not required for the study on human participants in accordance with the local legislation and institutional requirements. The participants provided their written informed consent to participate in this study.

## Author Contributions

The author confirms being the sole contributor of this work and has approved it for publication.

## Conflict of Interest

The author declares that the research was conducted in the absence of any commercial or financial relationships that could be construed as a potential conflict of interest.

## Footnotes

1 Their 2B experiment required a purely spatial representation, since the small finger movement of just pressing a button was required, but not many detail were given for this experiment, and moreover the left and right fingers were simultaneously used for the response, thus the near-far location was confounded with the left-right dimension.

2 http://www.r-project.org/

3 https://cran.r-project.org/package=lmerTest

4 For this analysis, given the distribution skewness (1.91) and excess kurtosis (6.43) values, data were log-transformed.

5 The formula is 217-(1,3^∗^S)-(0,6^∗^W), where *S* = number of syllables and *W* = number of words in the sentence. The mean Flesch index of no-transfer (N) and transfer (TR) sentences were compared (Means: *N* = 192.77, sd 1.07, TR = 192.59, sd = 0.99; *t*-test = 1.29, df = 238, *p* = 0.19). Bayes factor, also computed for *t*-test with the same data, was 6.195, showing substantial evidence for the null hypothesis.
