# Supplementary material for: Spatial and Motor Aspects in the “Action-Sentence Compatibility Effect”
Source: Front Psychol. 2021 Apr 9;12:647899. doi: 10.3389/fpsyg.2021.647899 (PMC8062728; doi:10.3389/fpsyg.2021.647899)
Supplement: Supplementary file 1 [file Data_Sheet_1.zip › Appendix.doc]

Spatial and motor aspects
in the “Action-Sentence Compatibility Effect”

Alberto Greco

Cognilab, Laboratory of Psychology and Cognitive Sciences, Dept. of Educational Sciences (DISFOR), University of Genoa, Genova (Italy)

**Correspondence:**
greco@unige.it

Keywords: action-sentence compatibility effect, ACE, embodiment, motor representation, spatial representation, action language

**Supplementary material** (referred in the text as Appendix)

**Legend**

variable names: RT = reaction times, sent_dir = sentence direction, R_dir = response direction, sent_code = sentence code

A,N,T = Away,No-transfer,Toward

## Table 1 Experiment 1

#### 1a - Exp 1 Outliers computed with the standard method

> data = read.csv2("exp1.csv", sep=";", header=T)

> moments::skewness(data$RT)

[1] 1.634032

> moments::kurtosis(data$RT)

[1] 7.35271
excess kurtosis = 4.35

#### 1b - Exp 1 Outliers computed with the extended method

```
> data = read.csv2("exp1_e.csv", sep=";", header=T)
> attach(data)
> moments::skewness(data$RT)
[1] 0.9567841
> moments::kurtosis(data$RT)
[1] 3.624076
excess kurtosis = .62
> LMM<-lmer(RT~type+block+group+sent_dir+R_dir+sent_dir*R_dir+(1|subj_code)+(1|sent_code),data=data)
> summary(LMM)
Linear mixed model fit by REML
t-tests use  Satterthwaite approximations to degrees of freedom ['lmerMod']
Formula: RT ~ type + block + group + sent_dir + R_dir + sent_dir * R_dir + (1 | subj_code) + (1 | sent_code)
   Data: data
REML criterion at convergence: 24582.7
Scaled residuals:
    Min      1Q  Median      3Q     Max
-2.1743 -0.6855 -0.1542  0.4792  3.9354
Random effects:
 Groups   Name        Variance Std.Dev.
 sent_code (Intercept)   8458    91.97
 subj_code  (Intercept)  77002   277.49
 Residual               136164   369.00
Number of obs: 1671, groups:  sent_code, 80; subj_code, 25
Fixed effects:
                   Estimate   Std. Error   df   t value   Pr(>|t|)
(Intercept)         1721.73      91.71   42.11  18.773  < 2e-16 ***
typeC                -28.40      27.41   76.54  -1.036   0.3035
block               -161.90      27.41   76.55  -5.906 9.07e-08 ***
groupB              -112.11     112.56   22.92  -0.996   0.3296
sent_dirV             80.76      32.69  153.05   2.471   0.0146 *
R_dirYN              -19.41      25.43 1582.95  -0.763   0.4454
sent_dirV:R_dirYN    -18.70      36.24 1585.55  -0.516   0.6059
---
Signif. codes:  0 ‘***’ 0.001 ‘**’ 0.01 ‘*’ 0.05 ‘.’ 0.1 ‘ ’ 1

> anova(LMM)
Analysis of Variance Table of type III  with  Satterthwaite
approximation for degrees of freedom
                        Sum Sq   Mean Sq  NumDF DenDF  F.value    Pr(>F)
type                     146152  146152     1   76.54   1.073   0.30345
block                   4750274 4750274     1   76.55  34.886   9.066e-08 ***
group                    135098  135098     1   22.92   0.992   0.32961
sent_dir                 925146  925146     1   76.39   6.794   0.01099 *
R_dir                    342589  342589     1 1610.12   2.516   0.11289
sent_dir:R_dir            36269   36269     1 1610.52   0.266   0.60585

> tapply(data$RT, list(data$block),mean)
   1        2
1507.412 1352.958
> tapply(data$RT, list(data$sent_dir),mean)
    A        T
1396.700 1465.531
> eta_sq(LMM, partial = TRUE)
          term      partial.etasq
1         type         0.014
2         block        0.313 large
3         group        0.041
4         sent_dir     0.082 medium
5         R_dir        0.002
6 sent_dir:R_dir       0.000
(Cohen recommend.: 0.01 = small, 0.06 = medium, 0.13 = large)
```

#### 1c - Exp 1 Separate analysis for Block 1

```
> block1 <- data[ which(block==1),]
> LMM<-lmer(RT~type+ sent_dir+R_dir+sent_dir*R_dir+(1|subj_code)+(1|sent_code),data=block1)

> summary(LMM)
Linear mixed model fit by REML. t-tests use Satterthwaite's method ['lmerModLmerTest']
Formula: RT ~ type + sent_dir + R_dir + sent_dir * R_dir + (1 | subj_code) + (1 | sent_code)
   Data: block1
REML criterion at convergence: 12364.2
Scaled residuals:
    Min      1Q  Median      3Q     Max
-2.2101 -0.6301 -0.1541  0.4621  3.5511
Random effects:
 Groups   Name        Variance Std.Dev.
 sent_code (Intercept)  10519   102.6
 subj_code  (Intercept)     92872   304.7
 Residual                  134169   366.3
Number of obs: 840, groups:  sent_code, 40; subj_code, 25
Fixed effects:
                          Estimate   Std. Error    df    t value   Pr(>|t|)
(Intercept)               1551.550     93.450   30.936  16.603   <2e-16 ***
typeC                      -28.238     41.220   37.351  -0.685   0.4975
sent_dirV                  100.543     48.076   68.516   2.091   0.0402 *
R_dirYN                   -140.147    127.079   24.803  -1.103   0.2807
sent_dirV:R_dirYN           -2.518     50.736  779.221  -0.050   0.9604

> anova(LMM)
Type III Analysis of Variance Table with Satterthwaite's method
                Sum Sq Mean Sq NumDF  DenDF F value  Pr(>F)
type              62967   62967     1  37.35  0.4693 0.49754
sent_dir         779941  779941     1  37.20  5.8131 0.02096 *
R_dir            172766  172766     1  22.94  1.2877 0.26820
sent_dir:R_dir      331     331     1 779.22  0.0025 0.96042
```

#### 1d - Exp 1 Separate analysis for Block 2

```
> block2 <- data[ which(block==2),]
> LMM<-lmer(RT~type+ sent_dir+R_dir+sent_dir*R_dir+(1|subj_code)+(1|sent_code),data=block2)

> summary(LMM)
Linear mixed model fit by REML. t-tests use Satterthwaite's method ['lmerModLmerTest']
Formula: RT ~ type + sent_dir + R_dir + sent_dir * R_dir + (1 | subj_code) + (1 | sent_code)
   Data: block2
REML criterion at convergence: 12185.1
Scaled residuals:
    Min      1Q  Median      3Q     Max
-1.8484 -0.6671 -0.2065  0.4445  4.3619
Random effects:
 Groups   Name        Variance Std.Dev.
 sent_code (Intercept)   6459    80.37
 subj_code (Intercept)  69670   263.95
 Residual              129092   359.29
Number of obs: 831, groups:  sent_code, 40; subj_code, 25
Fixed effects:
                   Estimate  Std. Error   df   t value    Pr(>|t|)
(Intercept)         1296.82      84.00   29.71  15.437 9.87e-16 ***
typeC                -28.34      35.67   37.26  -0.795    0.432
sent_dirV             59.29      43.40   80.59   1.366    0.176
R_dirYN               99.92     111.38   25.20   0.897    0.378
sent_dirV:R_dirYN    -31.50      50.06  774.51  -0.629    0.529

> anova(LMM)
Type III Analysis of Variance Table with Satterthwaite's method
                Sum Sq Mean Sq NumDF  DenDF F value Pr(>F)
type              81526   81526     1  37.26  0.6315 0.4318
sent_dir         192442  192442     1  37.22  1.4907 0.2298
R_dir             77526   77526     1  22.79  0.6005 0.4463
sent_dir:R_dir    51122   51122     1 774.51  0.3960 0.5293
```

#### 1e - Exp 1 Analysis including no-transfer sentences

```
> data = read.csv2("exp1_n", sep=";", header=T)
> tapply(data$RT, list(data$sent_dir),mean)
       A        N        T
1396.700 1495.958 1465.531

> LMM<-lmer (RT ~ type + block + group + sent_dir + R_dir + sent_dir * R_dir + (1 | subj_code) + (1 | sent_code), data=data)
Linear mixed model fit by REML. t-tests use Satterthwaite's method ['lmerModLmerTest']
Formula: RT ~ type + block + group + sent_dir + R_dir + sent_dir *      R_dir + (1 | subj_code) + (1 | sent_code)
   Data: data
REML criterion at convergence: 36454.4
Scaled residuals:
    Min      1Q  Median      3Q     Max
-2.2397 -0.6880 -0.1632  0.4715  3.9230
Random effects:
 Groups   Name        Variance Std.Dev.
 sent_code (Intercept)   9101    95.4
 subj_code (Intercept)  74467   272.9
 Residual               141223   375.8
Number of obs: 2473, groups:  sent_code, 120; subj_code, 25
Fixed effects:
                   Estimate   Std. Error   df    t value   Pr(>|t|)
(Intercept)        1761.619     87.656   39.055  20.097  < 2e-16 ***
typeC                -54.240     23.105  113.442  -2.348 0.020629 *
block               -183.562     23.109  113.518  -7.943 1.57e-12 ***
groupB              -103.368    110.293   22.955  -0.937 0.358407
sent_dirN            118.699     33.904  230.384   3.501 0.000556 ***
sent_dirV             81.575     33.531  221.923   2.433 0.015774 *
R_dirYN              -18.530     25.884 2338.901  -0.716 0.474135
sent_dirN:R_dirYN     -8.555     37.160 2344.021  -0.230 0.817935
sent_dirV:R_dirYN    -20.074     36.896 2342.634  -0.544 0.586436

> anova(LMM)
Type III Analysis of Variance Table with Satterthwaite's method
                 Sum Sq  Mean Sq  NumDF DenDF   F value    Pr(>F)
type              778293  778293     1  113.44  5.5111 0.0206293 *
block            8910444 8910444     1  113.52 63.0949 1.567e-12 ***
group             124045  124045     1   22.95  0.8784 0.3584070
sent_dir         2360684 1180342     2  113.53  8.3580 0.0004109 ***
R_dir             483024  483024     1 2344.43  3.4203 0.0645251 .
sent_dir:R_dir     42042   21021     2 2344.61  0.1488 0.8617069
---
Signif. codes:  0 ‘***’ 0.001 ‘**’ 0.01 ‘*’ 0.05 ‘.’ 0.1 ‘ ’ 1

> eta_sq(LMM, partial = TRUE)
            term     partial.etasq
1           type        0.046
2          block        0.357
3          group        0.037
4       sent_dir        0.128
5          R_dir        0.001
6 sent_dir:R_dir        0.000
```

#### 1f - Exp 1 Power simulations

> result1=powerCurve(LMM, test=fixed("block", method="anova"), along="subj_code", nsim=1000)

> print (result1)

Power for predictor 'block', (95% confidence interval),

by number of levels in subj_code:

3: 0.00% ( 0.00, 0.37) - 192 rows

5: 0.00% ( 0.00, 0.37) - 318 rows

8: 0.00% ( 0.00, 0.37) - 527 rows

10: 0.00% ( 0.00, 0.37) - 654 rows

13: 0.00% ( 0.00, 0.37) - 841 rows

15: 98.50% (97.54, 99.16) - 978 rows

18: 100.0% (99.63, 100.0) - 1179 rows

20: 100.0% (99.63, 100.0) - 1319 rows

23: 100.0% (99.63, 100.0) - 1529 rows

25: 100.0% (99.63, 100.0) - 1671 rows

result1=powerCurve(LMM, test=fixed("sent_dir", method="anova"), along="subj_code", nsim=1000, seed=99)

> print (result1)

Power for predictor 'sent_dir', (95% confidence interval),

by number of levels in subj_code:

3: 0.00% ( 0.00, 0.37) - 192 rows

5: 0.00% ( 0.00, 0.37) - 318 rows

8: 0.00% ( 0.00, 0.37) - 527 rows

10: 0.00% ( 0.00, 0.37) - 654 rows

13: 0.00% ( 0.00, 0.37) - 841 rows

15: 63.40% (60.33, 66.39) - 978 rows

18: 67.80% (64.80, 70.69) - 1179 rows

20: 71.30% (68.39, 74.09) - 1319 rows

23: 72.70% (69.82, 75.44) - 1529 rows

25: 74.20% (71.37, 76.89) - 1671 rows

### Table 2 - Experiment 2

#### 2a - Exp 2 Outliers computed with the extended method

```
> data = read.csv2("exp2.csv", sep=";", header=T)
> moments::skewness(data$RT)
[1] 0.9967277
> moments::kurtosis(data$RT)
[1] 3.93535

> LMM<-lmer(RT~type+block+group+sent_dir+R_dir+sent_dir*R_dir+(1|subj_code)+(1|sent_code),data=data)
Linear mixed model fit by REML. t-tests use Satterthwaite's method ['lmerModLmerTest']
Formula: RT ~ type + block + group + sent_dir + R_dir + sent_dir * R_dir + (1 | subj_code) + (1 | sent_code)
   Data: data
REML criterion at convergence: 23830.9
Scaled residuals:
    Min      1Q  Median      3Q     Max
-2.2910 -0.6629 -0.2079  0.4783  4.4416
Random effects:
 Groups   Name        Variance Std.Dev.
 sent_code (Intercept)   14471   120.3
 subj_code (Intercept)   35902   189.5
 Residual               126710   356.0
Number of obs: 1627, groups:  sent_code, 80; subj_code, 24
Fixed effects:
                   Estimate Std. Error      df t value Pr(>|t|)
(Intercept)         1684.28      80.02   69.39  21.049  < 2e-16 ***
typeC                -43.15      32.25   72.91  -1.338   0.1851
block               -151.39      32.22   72.87  -4.698 1.21e-05 ***
groupB              -133.55      79.36   22.00  -1.683   0.1065
sent_dirV             36.28      36.79  123.18   0.986   0.3260
R_dirYN               45.25      25.10 1526.25   1.803   0.0717 .
sent_dirV:R_dirYN    -39.50      35.43 1526.27  -1.115   0.2650
---
Signif. codes:  0 ‘***’ 0.001 ‘**’ 0.01 ‘*’ 0.05 ‘.’ 0.1 ‘ ’ 1

> anova(LMM)
Type III Analysis of Variance Table with Satterthwaite's method
                 Sum Sq Mean Sq NumDF   DenDF F value    Pr(>F)
type             226844  226844     1   72.91  1.7903    0.1851
block           2796718 2796718     1   72.87 22.0718 1.207e-05 ***
group            358859  358859     1   22.00  2.8321    0.1065
sent_dir          33330   33330     1   72.93  0.2630    0.6096
R_dir            262480  262480     1 1526.45  2.0715    0.1503
sent_dir:R_dir   157568  157568     1 1526.27  1.2435    0.2650
---
Signif. codes:  0 ‘***’ 0.001 ‘**’ 0.01 ‘*’ 0.05 ‘.’ 0.1 ‘ ’ 1

> tapply(data$RT, list(data$block),mean)
     1        2
1473.409 1318.312

> eta_sq(LMM, partial = TRUE)
             term  partial.etasq
1            type         0.024
2           block         0.232
3           group         0.114
4        sent_dir         0.004
5           R_dir         0.001
6  sent_dir:R_dir         0.001
```

#### 2b - Exp 2 Analysis including no-transfer sentences

```
> data = read.csv2("exp2_n.csv", sep=";", header=T)
> LMM<-lmer(RT~type+block+group+sent_dir+R_dir+sent_dir*R_dir+(1|subj_code)+(1|sent_code),data=data)

> summary(LMM)
Linear mixed model fit by REML. t-tests use Satterthwaite's method ['lmerModLmerTest']
Formula: RT ~ type + block + group + sent_dir + R_dir + sent_dir *      R_dir + (1 | subj_code) + (1 | sent_code)
   Data: data
REML criterion at convergence: 35256.9
Scaled residuals:
    Min      1Q  Median      3Q     Max
-2.3860 -0.6788 -0.1969  0.5141  4.4680
Random effects:
 Groups   Name        Variance Std.Dev.
 sent_code (Intercept)   15346   123.9
 subj_code (Intercept)   36603   191.3
 Residual               135379   367.9
Number of obs: 2397, groups:  sent_code, 120; subj_code, 24
Fixed effects:
                   Estimate Std. Error      df t value  Pr(>|t|)
(Intercept)         1708.46      75.63   63.96  22.590   < 2e-16 ***
typeC                -69.07      27.23  108.99  -2.536    0.0126 *
block               -165.31      27.22  108.98  -6.073  1.87e-08 ***
groupB              -113.74      79.55   21.96  -1.430    0.1668
sent_dirN            152.84      38.17  186.22   4.004  8.97e-05 ***
sent_dirV             35.75      37.94  182.29   0.942    0.3474
R_dirYN               46.72      25.94 2256.02   1.801    0.0718 .
sent_dirN:R_dirYN    -26.65      37.21 2261.00  -0.716    0.4739
sent_dirV:R_dirYN    -40.41      36.61 2256.04  -1.104    0.2697
---
Signif. codes:  0 ‘***’ 0.001 ‘**’ 0.01 ‘*’ 0.05 ‘.’ 0.1 ‘ ’ 1

> anova(LMM)
Type III Analysis of Variance Table with Satterthwaite's method
                 Sum Sq Mean Sq NumDF   DenDF F value    Pr(>F)
type            870995  870995     1  108.99  6.4338   0.01261 *
block          4992989 4992989     1  108.98 36.8817 1.870e-08 ***
group           276767  276767     1   21.96  2.0444   0.16684
sent_dir       2828559 1414280     2  108.98 10.4468 7.067e-05 ***
R_dir           352386  352386     1 2260.00  2.6030   0.10680
sent_dir:R_dir  170201   85100     2 2259.58  0.6286   0.53343
---
Signif. codes:  0 ‘***’ 0.001 ‘**’ 0.01 ‘*’ 0.05 ‘.’ 0.1 ‘ ’ 1

> tapply(data$RT, list(data$sent_dir),mean)
       A        N        T
1386.616 1512.662 1402.083

> eta_sq(LMM, partial = TRUE)
             term partial.etasq
1            type         0.056
2           block         0.253
3           group         0.085
4        sent_dir         0.161
5           R_dir         0.001
6  sent_dir:R_dir         0.001
```

#### 2c - Exp 2 Separate analysis for Block 1

```
> block1 <- data[ which(block==1),]
> LMM<-lmer(RT~type+ sent_dir+R_dir+sent_dir*R_dir+(1|subj_code)+(1|sent_code),data=block1)
> summary(LMM)
Linear mixed model fit by REML. t-tests use Satterthwaite's method ['lmerModLmerTest']
Formula: RT ~ type + sent_dir + R_dir + sent_dir * R_dir + (1 |      subj_code) + (1 | sent_code)
   Data: block1
REML criterion at convergence: 11926.8
Scaled residuals:
    Min      1Q  Median      3Q     Max
-2.1652 -0.6414 -0.1857  0.4654  3.9426
Random effects:
 Groups   Name        Variance Std.Dev.
 sent_code (Intercept)  21397   146.3
 subj_code (Intercept)  40099   200.2
 Residual              131261   362.3
Number of obs: 810, groups:  sent_code, 80; subj_code, 24
Fixed effects:
                   Estimate Std. Error      df t value Pr(>|t|)
(Intercept)         1391.13      60.50   77.96  22.995   <2e-16 ***
typeC                -30.51      41.84   77.78  -0.729    0.468
sent_dirV             63.34      51.98  173.50   1.219    0.225
R_dirYN               17.31      40.06  756.05   0.432    0.666
sent_dirV:R_dirYN    -60.81      53.65  749.32  -1.134    0.257
---
Signif. codes:  0 ‘***’ 0.001 ‘**’ 0.01 ‘*’ 0.05 ‘.’ 0.1 ‘ ’ 1

> anova(LMM)
Type III Analysis of Variance Table with Satterthwaite's method
                Sum Sq Mean Sq NumDF  DenDF F value  Pr(>F)
type              69785   69785     1  77.78  0.5317  0.4681
sent_dir          80608   80608     1  79.39  0.6141  0.4356
R_dir             31366   31366     1 751.82  0.2390  0.6251
sent_dir:R_dir   168663  168663     1 749.32  1.2849  0.2573
```

#### 2d - Exp 2 Separate analysis for Block 2

```
> block2 <- data[ which(block==2),]
> LMM<-lmer(RT~type+ sent_dir+R_dir+sent_dir*R_dir+(1|subj_code)+(1|sent_code),data=block2)
> summary(LMM)
Linear mixed model fit by REML. t-tests use Satterthwaite's method ['lmerModLmerTest']
Formula: RT ~ type + sent_dir + R_dir + sent_dir * R_dir + (1 |subj_code) + (1 | sent_code)
   Data: block2
REML criterion at convergence: 11970.9
Scaled residuals:
    Min      1Q  Median      3Q     Max
-2.0998 -0.6500 -0.1805  0.4991  4.4241
Random effects:
 Groups   Name        Variance Std.Dev.
 sent_code (Intercept)  17593   132.6
 subj_code  (Intercept)  37787   194.4
 Residual             122985   350.7
Number of obs: 817, groups:  sent_code, 80; subj_code, 24
Fixed effects:
                   Estimate Std. Error      df t value Pr(>|t|)
(Intercept)         1387.86      53.96   55.65  25.719   <2e-16 ***
typeC                -49.92      38.93   72.67  -1.282   0.2038
sent_dirV             17.91      44.89  124.85   0.399   0.6905
R_dirYN               71.41      34.83  753.09   2.050   0.0407 *
sent_dirV:R_dirYN    -32.25      51.03  748.24  -0.632   0.5276
---
Signif. codes:  0 ‘***’ 0.001 ‘**’ 0.01 ‘*’ 0.05 ‘.’ 0.1 ‘ ’ 1

> anova(LMM)
Type III Analysis of Variance Table with Satterthwaite's method
                Sum Sq Mean Sq NumDF  DenDF F value  Pr(>F)
type            202233  202233     1  72.67  1.6444 0.20380
sent_dir           259     259     1  73.47  0.0021 0.96349
R_dir           578219  578219     1 747.34  4.7015 0.03045 *
sent_dir:R_dir   49109   49109     1 748.24  0.3993 0.52764
---
Signif. codes:  0 ‘***’ 0.001 ‘**’ 0.01 ‘*’ 0.05 ‘.’ 0.1 ‘ ’ 1

> tapply(block2$RT, list(block2$R_dir),mean)
   YF       YN
1359.000 1420.806
```

#### 2e - Experiment 2 Power simulations

```
Exp 2 Power simulation (for Block)
> result1=powerCurve(LMM, test=fixed("block", method="anova"), along="subj_code", nsim=1000)
Calculating power at 10 sample sizes along subj_code
Power for predictor 'block', (95% confidence interval),
by number of levels in subj_code:
      3:  0.00% ( 0.00,  0.37) - 206 rows
      5:  0.00% ( 0.00,  0.37) - 344 rows
      8:  0.00% ( 0.00,  0.37) - 543 rows
     10:  0.00% ( 0.00,  0.37) - 682 rows
     12:  0.00% ( 0.00,  0.37) - 816 rows
     15: 96.90% (95.63, 97.88) - 1028 rows
     17: 98.80% (97.91, 99.38) - 1158 rows
     19: 99.50% (98.84, 99.84) - 1292 rows
     22: 99.70% (99.13, 99.94) - 1496 rows
     24: 99.60% (98.98, 99.89) - 1627 rows

Exp 2 Power simulation (for sentence direction)
> result1=powerCurve(LMM, test=fixed("sent_dir", method="anova"), along="subj_code", nsim=1000)
> print (result1)
Power for predictor 'sent_dir', (95% confidence interval),
by number of levels in subj_code:
      3:  0.00% ( 0.00,  0.37) - 290 rows
      5:  0.00% ( 0.00,  0.37) - 488 rows
      8:  0.00% ( 0.00,  0.37) - 780 rows
     10:  0.00% ( 0.00,  0.37) - 980 rows
     12:  0.00% ( 0.00,  0.37) - 1182 rows
     15: 96.70% (95.40, 97.72) - 1495 rows
     17: 97.40% (96.21, 98.29) - 1688 rows
     19: 97.60% (96.45, 98.46) - 1887 rows
     22: 98.30% (97.29, 99.01) - 2195 rows
     24: 98.70% (97.79, 99.31) - 2397 rows
```

### Table 3 - Experiment 3

#### 3a - Exp 3 Outliers computed with the extended method

```
> data = read.csv2("exp3.csv", sep=";", header=T)
> moments::skewness(data$RT)
[1] 0.9249874
> moments::kurtosis(data$RT)
[1] 3.744009

> LMM<-lmer(RT~type+block+group+sent_dir+R_dir+sent_dir*R_dir+(1|subj_code)+(1|sent_code),data=data)
> summary(LMM)
Linear mixed model fit by REML. t-tests use Satterthwaite's method ['lmerModLmerTest']
Formula: RT ~ type + block + group + sent_dir + R_dir + sent_dir * R_dir + (1 | subj_code) + (1 | sent_code)
   Data: data
REML criterion at convergence: 19581.8
Scaled residuals:
    Min      1Q  Median      3Q     Max
-2.1695 -0.6466 -0.1822  0.4628  4.1588
Random effects:
 Groups   Name        Variance Std.Dev.
 sent_code (Intercept)   13304   115.3
 subj_code  (Intercept)  50015   223.6
 Residual               115875   340.4
Number of obs: 1345, groups:  sent_code, 80; subj_code, 20
Fixed effects:
                   Estimate Std. Error      df t value Pr(>|t|)
(Intercept)         1744.62      91.54   43.27  19.059  < 2e-16 ***
typeC                -33.08      31.87   72.39  -1.038    0.303
block               -148.83      31.85   72.41  -4.673 1.34e-05 ***
groupB              -130.87     101.74   17.98  -1.286    0.215
sent_dirV            -24.69      36.91  129.63  -0.669    0.505
R_dirYN               19.18      26.29 1251.73   0.730    0.466
sent_dirV:R_dirYN     32.60      37.32 1252.33   0.873    0.383
---
Signif. codes:  0 ‘***’ 0.001 ‘**’ 0.01 ‘*’ 0.05 ‘.’ 0.1 ‘ ’ 1

> anova(LMM)
Type III Analysis of Variance Table with Satterthwaite's method
                 Sum Sq Mean Sq NumDF   DenDF F value    Pr(>F)
type             124855  124855     1   72.39  1.0775   0.30271
block           2530350 2530350     1   72.41 21.8368 1.337e-05 ***
group            191707  191707     1   17.98  1.6544   0.21467
sent_dir           8053    8053     1   72.37  0.0695   0.79282
R_dir            418469  418469     1 1251.65  3.6114   0.05762 .
sent_dir:R_dir    88401   88401     1 1252.33  0.7629   0.38259
---
Signif. codes:  0 ‘***’ 0.001 ‘**’ 0.01 ‘*’ 0.05 ‘.’ 0.1 ‘ ’ 1

> tapply(data$RT, list(data$block),mean)
       1        2
1509.643 1359.568

> eta_sq(LMM, partial = TRUE)
             term   partial.etasq
1            type        0.015
2           block        0.232
3           group        0.084
4        sent_dir        0.001
5           R_dir        0.003
6  sent_dir:R_dir        0.001
```

#### 3b - Exp 3 Separate analysis for Block 1

```
> block1 <- data[ which(block==1),]
> LMM<-lmer(RT~type+ sent_dir+R_dir+sent_dir*R_dir+(1|subj_code)+(1|sent_code),data=block1)
> summary(LMM)
Linear mixed model fit by REML. t-tests use Satterthwaite's method ['lmerModLmerTest']
Formula: RT ~ type + sent_dir + R_dir + sent_dir * R_dir + (1 |      subj_code) + (1 | sent_code)
   Data: block1
REML criterion at convergence: 9259.6
Scaled residuals:
    Min      1Q  Median      3Q     Max
-2.1317 -0.6272 -0.1434  0.4869  3.6560
Random effects:
 Groups   Name        Variance Std.Dev.
 sent_code (Intercept)  17506    132.3
 subj_code (Intercept)  57582    240.0
 Residual               97549    312.3
Number of obs: 641, groups:  sent_code, 80; subj_code, 20
Fixed effects:
                   Estimate Std. Error       df t value Pr(>|t|)
(Intercept)        1423.809     67.576   41.247  21.070   <2e-16 ***
typeC               -25.616     39.103   71.482  -0.655    0.515
sent_dirV             5.591     48.622  158.741   0.115    0.909
R_dirYN              46.695     39.269  591.521   1.189    0.235
sent_dirV:R_dirYN   -21.340     52.074  587.262  -0.410    0.682
---
Signif. codes:  0 ‘***’ 0.001 ‘**’ 0.01 ‘*’ 0.05 ‘.’ 0.1 ‘ ’ 1

> anova(LMM)
Type III Analysis of Variance Table with Satterthwaite's method
                Sum Sq Mean Sq NumDF  DenDF F value Pr(>F)
type             41861   41861     1  71.48  0.4291 0.5145
sent_dir          1629    1629     1  73.45  0.0167 0.8975
R_dir           187032  187032     1 587.94  1.9173 0.1667
sent_dir:R_dir   16382   16382     1 587.26  0.1679 0.6821
```

#### 3c - Exp 3 Separate analysis for Block 2

```
> block2 <- data[ which(block==2),]
> LMM<-lmer(RT~type+ sent_dir+R_dir+sent_dir*R_dir+(1|subj_code)+(1|sent_code),data=block2)
> summary(LMM)
Linear mixed model fit by REML. t-tests use Satterthwaite's method ['lmerModLmerTest']
Formula: RT ~ type + sent_dir + R_dir + sent_dir * R_dir + (1 |      subj_code) + (1 | sent_code)
   Data: block2
REML criterion at convergence: 10364.3
Scaled residuals:
    Min      1Q  Median      3Q     Max
-1.9386 -0.6424 -0.1904  0.4330  3.5848


Random effects:
 Groups   Name        Variance Std.Dev.
 sent_code (Intercept)  21081   145.2
 subj_code (Intercept)  45017   212.2
 Residual              131813   363.1
Number of obs: 704, groups:  sent_code, 80; subj_code, 20
Fixed effects:
                   Estimate Std. Error      df t value Pr(>|t|)
(Intercept)         1465.89      62.63   44.90  23.404   <2e-16 ***
typeC                -36.09      43.17   73.36  -0.836    0.406
sent_dirV            -31.90      50.35  132.68  -0.634    0.527
R_dirYN                9.08      37.38  631.00   0.243    0.808
sent_dirV:R_dirYN     65.48      57.22  644.27   1.144    0.253
---
Signif. codes:  0 ‘***’ 0.001 ‘**’ 0.01 ‘*’ 0.05 ‘.’ 0.1 ‘ ’ 1

> anova(LMM)
Type III Analysis of Variance Table with Satterthwaite's method
                Sum Sq Mean Sq NumDF  DenDF F value Pr(>F)
type             92125   92125     1  73.37  0.6989 0.4059
sent_dir            50      50     1  75.38  0.0004 0.9845
R_dir           284214  284214     1 642.56  2.1562 0.1425
sent_dir:R_dir  172616  172616     1 644.27  1.3096 0.2529
```

#### 3d - Exp 3 Analysis including no-transfer sentences

```
> data = read.csv2("exp3_n.csv", sep=";", header=T)
> tapply(data$RT, list(data$sent_dir),mean)
       A        N        T
1435.449 1614.272 1432.515

> LMM<-lmer(RT~type+block+group+sent_dir+R_dir+sent_dir*R_dir+(1|subj_code)+(1|sent_code),data=data)
> summary(LMM)
Linear mixed model fit by REML. t-tests use Satterthwaite's method ['lmerModLmerTest']
Formula: RT ~ type + block + group + sent_dir + R_dir + sent_dir * R_dir + (1 | subj_code) + (1 | sent_code)
   Data: data
REML criterion at convergence: 29096.4
Scaled residuals:
    Min      1Q  Median      3Q     Max
-2.3599 -0.6759 -0.1771  0.5032  4.1564
Random effects:
 Groups   Name        Variance Std.Dev.
 sent_code (Intercept)  14889   122.0
 subj_code (Intercept)  50070   223.8
 Residual              129618   360.0
Number of obs: 1984, groups:  sent_code, 120; subj_code, 20
Fixed effects:
                   Estimate Std. Error       df t value Pr(>|t|)
(Intercept)        1737.220     88.271   39.724  19.680  < 2e-16 ***
typeC               -58.148     27.616  107.792  -2.106   0.0376 *
block              -132.396     27.604  107.793  -4.796 5.22e-06 ***
groupB             -138.764    101.385   17.987  -1.369   0.1880
sent_dirN           182.410     39.259  194.220   4.646 6.22e-06 ***
sent_dirV           -25.579     39.034  190.711  -0.655   0.5131
R_dirYN              19.466     27.800 1851.773   0.700   0.4839
sent_dirN:R_dirYN     8.638     39.935 1854.714   0.216   0.8288
sent_dirV:R_dirYN    31.607     39.460 1852.506   0.801   0.4232
---
Signif. codes:  0 ‘***’ 0.001 ‘**’ 0.01 ‘*’ 0.05 ‘.’ 0.1 ‘ ’ 1

> anova(LMM)
Type III Analysis of Variance Table with Satterthwaite's method
                 Sum Sq Mean Sq NumDF   DenDF F value    Pr(>F)
type             574677  574677     1  107.79  4.4336   0.03756 *
block           2981721 2981721     1  107.79 23.0039 5.217e-06 ***
group            242813  242813     1   17.99  1.8733   0.18795
sent_dir        5520223 2760111     2  107.80 21.2942 1.608e-08 ***
R_dir            529765  529765     1 1853.98  4.0871   0.04335 *
sent_dir:R_dir    88491   44246     2 1854.13  0.3414   0.71085

> eta_sq(LMM, partial = TRUE)
             term partial.etasq
1            type         0.040
2           block         0.176
3           group         0.094
4        sent_dir         0.283
5           R_dir         0.002
6  sent_dir:R_dir         0.000

> tapply(data$RT, list(data$type),mean)
       A        C
1518.332 1467.122

> mod2<-lmer(RT~type+block+sent_dir+ type*sent_dir+(1|subj_code)+(1|sent_code),data=data)
> summary(mod2)
Linear mixed model fit by REML. t-tests use Satterthwaite's method ['lmerModLmerTest']
Formula: RT ~ type + block + sent_dir + type * sent_dir + (1 |  subj_code) + (1 | sent_code)
   Data: data
REML criterion at convergence: 29330.9
Scaled residuals:
    Min      1Q  Median      3Q     Max
-2.1887 -0.6116 -0.1419  0.3923  6.9960
Random effects:
 Groups   Name        Variance Std.Dev.
 sent_code (Intercept)  14083   118.7
 subj_code (Intercept) 126752   356.0
 Residual              221252   470.4
Number of obs: 1929, groups:  sent_code, 80; subj_code, 25
Fixed effects:
               Estimate Std. Error       df t value Pr(>|t|)
(Intercept)    1816.849     94.070   59.730  19.314  < 2e-16 ***
typeC           -79.217     48.196   74.582  -1.644   0.1045
block          -213.486     34.115   74.881  -6.258 2.21e-08 ***
sent_dirV        91.184     48.203   74.638   1.892   0.0624 .
typeC:sent_dirV   8.622     68.227   74.864   0.126   0.8998
---
Signif. codes:  0 ‘***’ 0.001 ‘**’ 0.01 ‘*’ 0.05 ‘.’ 0.1 ‘ ’ 1

> anova(mod2)
Type III Analysis of Variance Table with Satterthwaite's method
              Sum Sq Mean Sq NumDF  DenDF F value    Pr(>F)
type          1066820 1066820     1 74.857  4.8217  0.031201 *
block         8664122 8664122     1 74.881 39.1595 2.215e-08 ***
sent_dir      1733879 1733879     1 74.861  7.8367  0.006508 **
type:sent_dir    3534    3534     1 74.864  0.0160  0.899770
```

#### 3e - Exp 3 Power simulations

```
> result1=powerCurve(LMM, test=fixed("block", method="anova"), along="subj_code", nsim=1000)
> print(result1)
Power for predictor 'block', (95% confidence interval),
by number of levels in subj_code:
      3:  0.00% ( 0.00,  0.37) - 224 rows
      5:  0.00% ( 0.00,  0.37) - 379 rows
      8:  0.00% ( 0.00,  0.37) - 608 rows
     10:  0.00% ( 0.00,  0.37) - 766 rows
     13:  0.00% ( 0.00,  0.37) - 995 rows
     15: 99.10% (98.30, 99.59) - 1150 rows
     18: 100.0% (99.63, 100.0) - 1382 rows
     20: 100.0% (99.63, 100.0) - 1536 rows
     23: 100.0% (99.63, 100.0) - 1770 rows
     25: 100.0% (99.63, 100.0) - 1929 rows
result1=powerCurve(LMM, test=fixed("sent_dir", method="anova"), along="subj_code", nsim=1000)
> print(result1)
Power for predictor 'sent_dir', (95% confidence interval),
by number of levels in subj_code:
      3:  0.00% ( 0.00,  0.37) - 224 rows
      5:  0.00% ( 0.00,  0.37) - 379 rows
      8:  0.00% ( 0.00,  0.37) - 608 rows
     10:  0.00% ( 0.00,  0.37) - 766 rows
     13:  0.00% ( 0.00,  0.37) - 995 rows
     15: 67.30% (64.29, 70.20) - 1150 rows
     18: 72.60% (69.72, 75.34) - 1382 rows
     20: 74.40% (71.58, 77.08) - 1536 rows
     23: 75.40% (72.61, 78.04) - 1770 rows
     25: 78.10% (75.41, 80.63) - 1929 rows
result1=powerCurve(LMM, test=fixed("type", method="anova"), along="subj_code", nsim=1000)
```

### Table 4 - Experiment 4

#### 4a - Exp 4 Outliers computed with the extended method

```
> data = read.csv2("exp4.csv", sep=";", header=T)
> moments::skewness(data$RT)
[1] 0.7437735
> moments::kurtosis(data$RT)
[1] 3.011354

> tapply(data$RT, list(data$block),mean)
       1        2
1736.542 1563.565

> LMM<-lmer(RT~type+block+group+sent_dir+R_dir+sent_dir*R_dir+(1|subj_code)+(1|sent_code),data=data)
> summary(LMM)
Linear mixed model fit by REML. t-tests use Satterthwaite's method ['lmerModLmerTest']
Formula: RT ~ type + block + group + sent_dir + R_dir + sent_dir * R_dir + (1 | subj_code) + (1 | sent_code)
   Data: data
REML criterion at convergence: 19502
Scaled residuals:
    Min      1Q  Median      3Q     Max
-2.4347 -0.6928 -0.1767  0.5458  3.9445
Random effects:
 Groups   Name        Variance Std.Dev.
 sent_code (Intercept)   7399    86.01
 subj_code (Intercept)  68322   261.39
 Residual              162202   402.74
Number of obs: 1312, groups:  sent_code, 80; subj_code, 20
Fixed effects:
                   Estimate Std. Error      df t value Pr(>|t|)
(Intercept)         1846.73      98.99   32.86  18.656  < 2e-16 ***
typeC                -35.24      29.49   72.27  -1.195    0.236
block               -178.31      29.49   72.32  -6.046 5.92e-08 ***
groupB               160.67     119.04   17.77   1.350    0.194
sent_dirV             35.77      36.77  170.89   0.973    0.332
R_dirYN               10.93      31.94 1224.97   0.342    0.732
sent_dirV:R_dirYN     25.45      44.72 1226.81   0.569    0.569
---
Signif. codes:  0 ‘***’ 0.001 ‘**’ 0.01 ‘*’ 0.05 ‘.’ 0.1 ‘ ’ 1

> anova(LMM)
Type III Analysis of Variance Table with Satterthwaite's method
                 Sum Sq Mean Sq NumDF   DenDF F value    Pr(>F)
type             231614  231614     1   72.27  1.4279    0.2360
block           5929563 5929563     1   72.32 36.5568 5.917e-08 ***
group            295513  295513     1   17.77  1.8219    0.1940
sent_dir         438883  438883     1   72.27  2.7058    0.1043
R_dir            181754  181754     1 1223.46  1.1205    0.2900
sent_dir:R_dir    52550   52550     1 1226.81  0.3240    0.5693
---
Signif. codes:  0 ‘***’ 0.001 ‘**’ 0.01 ‘*’ 0.05 ‘.’ 0.1 ‘ ’ 1

> eta_sq(LMM, partial = TRUE)
             term partial.etasq
1            type         0.019
2           block         0.336
3           group         0.093
4        sent_dir         0.036
5           R_dir         0.001
6  sent_dir:R_dir         0.000
```

#### 4b - Exp 4 Separate analysis for Block 1

```
> block1 <- data[ which(block==1),]
> LMM<-lmer(RT~type+ sent_dir+R_dir+sent_dir*R_dir+(1|subj_code)+(1|sent_code),data=block1)
> summary(LMM)
Linear mixed model fit by REML. t-tests use Satterthwaite's method ['lmerModLmerTest']
Formula: RT ~ type + sent_dir + R_dir + sent_dir * R_dir + (1 |      subj_code) + (1 | sent_code)
   Data: block1
REML criterion at convergence: 9239.6
Scaled residuals:
    Min      1Q  Median      3Q     Max
-2.2423 -0.6996 -0.1698  0.5462  3.2206
Random effects:
 Groups   Name        Variance Std.Dev.
 sent_code (Intercept)  16681   129.2
 subj_code (Intercept)  70309   265.2
 Residual              157198   396.5
Number of obs: 621, groups:  sent_code, 80; subj_code, 20
Fixed effects:
                   Estimate Std. Error      df t value Pr(>|t|)
(Intercept)         1693.21      73.40   35.66  23.069   <2e-16 ***
typeC                -56.75      43.59   72.68  -1.302   0.1971
sent_dirV             24.77      54.19  158.05   0.457   0.6482
R_dirYN              -96.61      46.86  560.27  -2.062   0.0397 *
sent_dirV:R_dirYN     76.66      65.79  563.07   1.165   0.2444
---
Signif. codes:  0 ‘***’ 0.001 ‘**’ 0.01 ‘*’ 0.05 ‘.’ 0.1 ‘ ’ 1

> anova(LMM)
Type III Analysis of Variance Table with Satterthwaite's method
                Sum Sq Mean Sq NumDF  DenDF F value  Pr(>F)
type            266434  266434     1  72.68  1.6949 0.19707
sent_dir        327511  327511     1  73.13  2.0834 0.15318
R_dir           493215  493215     1 565.38  3.1375 0.07705 .
sent_dir:R_dir  213415  213415     1 563.07  1.3576 0.24444
---
Signif. codes:  0 ‘***’ 0.001 ‘**’ 0.01 ‘*’ 0.05 ‘.’ 0.1 ‘ ’ 1

> tapply(block1$RT, list(block1$R_dir),mean)
     YF       YN
1649.808 1601.175
```

#### 4c - Exp 4 Separate analysis for Block 2

```
> block2 <- data[ which(block==2),]
> LMM<-lmer(RT~type+ sent_dir+R_dir+sent_dir*R_dir+(1|subj_code)+(1|sent_code),data=block2)
> summary(LMM)
Linear mixed model fit by REML. t-tests use Satterthwaite's method ['lmerModLmerTest']
Formula: RT ~ type + sent_dir + R_dir + sent_dir * R_dir + (1 |      subj_code) + (1 | sent_code)
   Data: block2
REML criterion at convergence: 10304.4
Scaled residuals:
    Min      1Q  Median      3Q     Max
-2.2979 -0.6711 -0.1233  0.5476  3.5899
Random effects:
 Groups   Name        Variance Std.Dev.
 sent_code (Intercept)  14682   121.2
 subj_code (Intercept)  72437   269.1
 Residual              163726   404.6
Number of obs: 691, groups:  sent_code, 80; subj_code, 20
Fixed effects:
                   Estimate Std. Error      df t value Pr(>|t|)
(Intercept)         1605.81      74.08   36.09  21.677   <2e-16 ***
typeC                -20.52      41.59   76.33  -0.493   0.6231
sent_dirV             78.19      52.03  171.98   1.503   0.1347
R_dirYN              102.13      45.27  631.23   2.256   0.0244 *
sent_dirV:R_dirYN    -39.71      62.89  629.84  -0.631   0.5280
---
Signif. codes:  0 ‘***’ 0.001 ‘**’ 0.01 ‘*’ 0.05 ‘.’ 0.1 ‘ ’ 1

> anova(LMM)
Type III Analysis of Variance Table with Satterthwaite's method
                 Sum Sq Mean Sq NumDF  DenDF F value  Pr(>F)
type              39867   39867     1  76.33  0.2435 0.62311
sent_dir         323394  323394     1  75.69  1.9752 0.16399
R_dir           1115296 1115296     1 631.24  6.8120 0.00927 **
sent_dir:R_dir    65274   65274     1 629.84  0.3987 0.52800
---
Signif. codes:  0 ‘***’ 0.001 ‘**’ 0.01 ‘*’ 0.05 ‘.’ 0.1 ‘ ’ 1


> tapply(block2$RT, list(block2$R_dir),mean)
     YF       YN
1359.000 1420.806
```

#### 4d - Exp 4 Power simulation

```
> result1=powerCurve(LMM, test=fixed("block", method="anova"), along="subj_code", nsim=1000)
> print(result1)
Power for predictor 'block', (95% confidence interval),
by number of levels in subj_code:
      3:  0.00% ( 0.00,  0.37) - 198 rows
      5:  0.00% ( 0.00,  0.37) - 334 rows
      7:  0.00% ( 0.00,  0.37) - 476 rows
      9:  0.00% ( 0.00,  0.37) - 616 rows
     11: 87.60% (85.40, 89.58) - 748 rows
     12: 95.70% (94.25, 96.87) - 789 rows
     14: 99.50% (98.84, 99.84) - 906 rows
     16: 100.0% (99.63, 100.0) - 1047 rows
     18: 100.0% (99.63, 100.0) - 1182 rows
     20: 100.0% (99.63, 100.0) - 1312 rows
```

#### 4e - Exp 4 Analysis including no-transfer sentences

```
> data = read.csv2("exp4_n.csv", sep=";", header=T)
> tapply(data$RT, list(data$sent_dir),mean)
       A        N        T
1624.998 1799.665 1671.549

> LMM<-lmer(RT~type+block+group+sent_dir+R_dir+sent_dir*R_dir+(1|subj_code)+(1|sent_code),data=data)
> summary(LMM)
Linear mixed model fit by REML. t-tests use Satterthwaite's method ['lmerModLmerTest']
Formula: RT ~ type + block + group + sent_dir + R_dir + sent_dir * R_dir + (1 | subj_code) + (1 | sent_code)
   Data: data
REML criterion at convergence: 28208.9
Scaled residuals:
    Min      1Q  Median      3Q     Max
-2.3700 -0.7034 -0.1703  0.5561  3.9897
Random effects:
 Groups   Name        Variance Std.Dev.
 sent_code (Intercept)  10064   100.3
 subj_code (Intercept)  61899   248.8
 Residual              166758   408.4
Number of obs: 1894, groups:  sent_code, 120; subj_code, 20
Fixed effects:
                   Estimate Std. Error      df t value Pr(>|t|)
(Intercept)         1871.63      93.69   33.06  19.977  < 2e-16 ***
typeC                -63.24      26.36  105.10  -2.399   0.0182 *
block               -182.31      26.37  105.22  -6.914 3.77e-10 ***
groupB               150.00     112.88   17.80   1.329   0.2007
sent_dirN            190.01      39.84  238.95   4.770 3.21e-06 ***
sent_dirV             35.71      38.90  221.68   0.918   0.3597
R_dirYN               10.93      32.38 1767.33   0.338   0.7356
sent_dirN:R_dirYN     -3.24      46.91 1768.47  -0.069   0.9449
sent_dirV:R_dirYN     25.34      45.33 1769.48   0.559   0.5762
---
Signif. codes:  0 ‘***’ 0.001 ‘**’ 0.01 ‘*’ 0.05 ‘.’ 0.1 ‘ ’ 1

> anova(LMM)
Type III Analysis of Variance Table with Satterthwaite's method
                 Sum Sq Mean Sq NumDF   DenDF F value    Pr(>F)
type             959960  959960     1  105.10  5.7566   0.01819 *
block           7972088 7972088     1  105.22 47.8063 3.767e-10 ***
group            294496  294496     1   17.80  1.7660   0.20066
sent_dir        5970844 2985422     2  105.32 17.9027 2.029e-07 ***
R_dir            156482  156482     1 1765.70  0.9384   0.33283
sent_dir:R_dir    78227   39114     2 1769.36  0.2346   0.79095

> eta_sq(LMM, partial = TRUE)
             term partial.etasq
1            type         0.052
2           block         0.312
3           group         0.090
4        sent_dir         0.254
5           R_dir         0.001
6  sent_dir:R_dir         0.000

> result1=powerCurve(LMM, test=fixed("sent_dir", method="anova"), along="subj_code", nsim=1000)
> print(result1)
Power for predictor 'sent_dir', (95% confidence interval),
by number of levels in subj_code:
      3:  0.00% ( 0.00,  0.37) - 282 rows
      5:  0.00% ( 0.00,  0.37) - 480 rows
      7:  0.00% ( 0.00,  0.37) - 683 rows
      9:  0.00% ( 0.00,  0.37) - 884 rows
     11: 99.30% (98.56, 99.72) - 1082 rows
     12: 99.50% (98.84, 99.84) - 1138 rows
     14: 99.80% (99.28, 99.98) - 1308 rows
     16: 99.80% (99.28, 99.98) - 1517 rows
     18: 99.90% (99.44, 100.00) - 1707 rows
     20: 100.0% (99.63, 100.0) - 1894 rows


> tapply(data$RT, list(data$type),mean)
      A        C
1723.791 1667.389

> mod2<-lmer(RT~type+block+sent_dir+ type*sent_dir+(1|subj_code)+(1|sent_code),data=data)
> summary(mod2)
Linear mixed model fit by REML. t-tests use Satterthwaite's method ['lmerModLmerTest']
Formula: RT ~ type + block + sent_dir + type * sent_dir + (1 | subj_code) + (1 | sent_code)
   Data: data
REML criterion at convergence: 28227.4
Scaled residuals:
    Min      1Q  Median      3Q     Max
-2.4389 -0.7023 -0.1741  0.5546  4.0130
Random effects:
 Groups   Name        Variance Std.Dev.
 sent_code (Intercept)  10005   100.0
 subj_code (Intercept)  64452   253.9
 Residual              166607   408.2
Number of obs: 1894, groups:  sent_code, 120; subj_code, 20
Fixed effects:
               Estimate Std. Error      df t value Pr(>|t|)
(Intercept)      1936.45      76.28   51.98  25.385  < 2e-16 ***
typeC             -33.77      45.24  101.50  -0.746    0.457
block            -182.01      26.31  102.94  -6.917 4.01e-10 ***
sent_dirN         234.46      46.56  110.36   5.035 1.87e-06 ***
sent_dirV          50.24      45.06   99.68   1.115    0.268
typeC:sent_dirN   -89.66      65.08  106.50  -1.378    0.171
typeC:sent_dirV    -2.71      63.66   99.40  -0.043    0.966

> anova(mod2)
Type III Analysis of Variance Table with Satterthwaite's method
              Sum Sq Mean Sq NumDF  DenDF F value    Pr(>F)
type          1001653 1001653     1 103.33  6.0121   0.01588 *
block         7971025 7971025     1 102.94 47.8432 4.011e-10 ***
sent_dir      6057960 3028980     2 103.22 18.1803 1.722e-07 ***
type:sent_dir  406011  203006     2 103.28  1.2185   0.29990
```

### Table 5 - Experiment 5

#### 5a - Exp 5 Outliers computed with the extended method

```
> data = read.csv2("exp5.csv", sep=";", header=T)
> moments::skewness(data$RT)
[1] 0.5927235
> moments::kurtosis(data$RT)
[1] 2.715766

> tapply(data$RT, list(data$block),mean)
       1        2
1721.919 1603.503

> LMM<- lmer(RT~type+block+group+sent_dir+R_dir+sent_dir*R_dir+(1|subj_code)+(1|sent_code),data=data)
> summary(LMM)
Linear mixed model fit by REML. t-tests use Satterthwaite's method ['lmerModLmerTest']
Formula: RT ~ type + block + group + sent_dir + R_dir + sent_dir * R_dir + (1 | subj_code) + (1 | sent_code)
   Data: data
REML criterion at convergence: 24521.8
Scaled residuals:
    Min      1Q  Median      3Q     Max
-2.6273 -0.7057 -0.2140  0.5891  4.2397
Random effects:
 Groups   Name        Variance Std.Dev.
 sent_code (Intercept)  13641   116.8
 subj_code (Intercept)  61067   247.1
 Residual              179517   423.7
Number of obs: 1636, groups:  sent_code, 80; subj_code, 28
Fixed effects:
                   Estimate Std. Error       df t value Pr(>|t|)
(Intercept)        1823.482     88.826   71.433  20.529  < 2e-16 ***
typeC               -54.042     33.662   74.045  -1.605 0.112658
block              -121.959     33.644   74.084  -3.625 0.000527 ***
groupB                9.773     96.089   25.927   0.102 0.919775
sent_dirV            25.259     39.929  145.165   0.633 0.527992
R_dirYN              86.294     29.943 1544.222   2.882 0.004007 **
sent_dirV:R_dirYN    -6.059     42.245 1549.218  -0.143 0.885967

> anova (LMM)
Type III Analysis of Variance Table with Satterthwaite's method
                 Sum Sq Mean Sq NumDF   DenDF F value    Pr(>F)
type             462684  462684     1   74.04  2.5774 0.1126582
block           2358947 2358947     1   74.08 13.1405 0.0005273 ***
group              1857    1857     1   25.93  0.0103 0.9197753
sent_dir          78458   78458     1   73.89  0.4371 0.5106054
R_dir           2785824 2785824     1 1544.51 15.5184 8.535e-05 ***
sent_dir:R_dir     3693    3693     1 1549.22  0.0206 0.8859668
Signif. codes:  0 ‘***’ 0.001 ‘**’ 0.01 ‘*’ 0.05 ‘.’ 0.1 ‘ ’ 1

> eta_sq(LMM, partial = TRUE)
             term partial.etasq
1            type         0.034
2           block         0.151
3           group         0.000
4        sent_dir         0.006
5           R_dir         0.010
6  sent_dir:R_dir         0.000

> tapply(data$RT, list(data$R_dir),mean)
      YF       YN
1619.489 1702.046
```

#### 5b - Exp 5 Separate analysis for Block 1

```
> block1 <- data[ which(block==1),]
> LMM<-lmer(RT~type+ sent_dir+R_dir+sent_dir*R_dir+(1|subj_code)+(1|sent_code),data=block1)
> summary(LMM)
Linear mixed model fit by REML. t-tests use Satterthwaite's method ['lmerModLmerTest']
Formula: RT ~ type + sent_dir + R_dir + sent_dir * R_dir + (1 | subj_code) + (1 | sent_code)
   Data: block1
REML criterion at convergence: 12286.3
Scaled residuals:
    Min      1Q  Median      3Q     Max
-2.4810 -0.7231 -0.1964  0.5838  3.2835
Random effects:
 Groups   Name        Variance Std.Dev.
 sent_code (Intercept)   7888    88.81
 subj_code (Intercept)  55162   234.87
 Residual              189850   435.72
Number of obs: 817, groups:  sent_code, 80; subj_code, 28
Fixed effects:
                   Estimate Std. Error      df t value Pr(>|t|)
(Intercept)          1632.90      58.85   58.28  27.749   <2e-16 ***
typeC                 -26.10      36.98   67.65  -0.706   0.4828
sent_dirV              36.16      49.03  190.38   0.737   0.4617
R_dirYN               115.17      45.07  778.01   2.555   0.0108 *
sent_dirV:R_dirYN     -85.28      63.10  776.07  -1.352   0.1769
---
Signif. codes:  0 ‘***’ 0.001 ‘**’ 0.01 ‘*’ 0.05 ‘.’ 0.1 ‘ ’ 1

> anova(LMM)
Type III Analysis of Variance Table with Satterthwaite's method
                 Sum Sq Mean Sq NumDF  DenDF F value  Pr(>F)
type              94545   94545     1  67.65  0.4980 0.48280
sent_dir           5734    5734     1  69.71  0.0302 0.86254
R_dir           1005872 1005872     1 774.14  5.2982 0.02161 *
sent_dir:R_dir   346790  346790     1 776.07  1.8267 0.17692
---
Signif. codes:  0 ‘***’ 0.001 ‘**’ 0.01 ‘*’ 0.05 ‘.’ 0.1 ‘ ’ 1

> tapply(block1$RT, list(block1$R_dir),mean)
      YF       YN
1623.729 1693.694
```

#### 5c - Exp 5 Separate analysis for Block 2

```
> block2 <- data[ which(block==2),]
> LMM<-lmer(RT~type+ sent_dir+R_dir+sent_dir*R_dir+(1|subj_code)+(1|sent_code),data=block2)
> summary(LMM)
Linear mixed model fit by REML. t-tests use Satterthwaite's method ['lmerModLmerTest']
Formula: RT ~ type + sent_dir + R_dir + sent_dir * R_dir + (1 |      subj_code) + (1 | sent_code)
   Data: block2
REML criterion at convergence: 12288.5
Scaled residuals:
    Min      1Q  Median      3Q     Max
-2.3555 -0.6912 -0.1810  0.5513  4.1150
Random effects:
 Groups   Name        Variance Std.Dev.
 sent_code (Intercept)  20535   143.3
 subj_code (Intercept)  62156   249.3
 Residual              175234   418.6
Number of obs: 819, groups:  sent_code, 80; subj_code, 28
Fixed effects:
                   Estimate Std. Error      df t value Pr(>|t|)
(Intercept)          1652.71      66.25   75.87  24.945   <2e-16 ***
typeC                 -88.91      44.11   76.30  -2.016   0.0473 *
sent_dirV              22.36      54.06  161.44   0.414   0.6798
R_dirYN                66.64      42.84  759.46   1.556   0.1202
sent_dirV:R_dirYN      58.13      61.38  767.42   0.947   0.3439
---
Signif. codes:  0 ‘***’ 0.001 ‘**’ 0.01 ‘*’ 0.05 ‘.’ 0.1 ‘ ’ 1


> anova(LMM)
Type III Analysis of Variance Table with Satterthwaite's method
                 Sum Sq Mean Sq NumDF  DenDF F value   Pr(>F)
type             712013  712013     1  76.30  4.0632 0.047348 *
sent_dir         236394  236394     1  77.50  1.3490 0.249014
R_dir           1712541 1712541     1 766.44  9.7729 0.001838 **
sent_dir:R_dir   157195  157195     1 767.42  0.8971 0.343871

> tapply(block2$RT, list(block2$type),mean)
       A        C
1698.554 1632.054

> tapply(block2$RT, list(block2$R_dir),mean)
      YF       YN
1615.281 1710.417
```

#### 5d - Exp 5 Analysis including no-transfer sentences

```
> data = read.csv2("exp5_n.csv", sep=";", header=T)
> tapply(data$RT, list(data$sent_dir),mean)
       A        N        T
1656.251 1813.599 1666.934

> LMM<-lmer(RT~type+block+group+sent_dir+R_dir+sent_dir*R_dir+(1|subj_code)+(1|sent_code),data=data)
> summary(LMM)
Linear mixed model fit by REML. t-tests use Satterthwaite's method ['lmerModLmerTest']
Formula: RT ~ type + block + group + sent_dir + R_dir + sent_dir *      R_dir + (1 | subj_code) + (1 | sent_code)
   Data: data
REML criterion at convergence: 35505.8
Scaled residuals:
    Min      1Q  Median      3Q     Max
-2.5187 -0.7106 -0.1923  0.5913  4.1662
Random effects:
 Groups   Name        Variance Std.Dev.
 sent_code (Intercept)  13626   116.7
 subj_code (Intercept)  64238   253.5
 Residual              182634   427.4
Number of obs: 2367, groups:  sent_code, 120; subj_code, 28
Fixed effects:
                   Estimate Std. Error       df t value Pr(>|t|)
(Intercept)        1808.273     84.622   62.753  21.369  < 2e-16 ***
typeC               -66.946     27.819  114.178  -2.406 0.017712 *
block              -110.377     27.821  114.424  -3.967 0.000127 ***
groupB               21.391     97.738   25.925   0.219 0.828472
sent_dirN           194.054     40.832  232.380   4.753 3.52e-06 ***
sent_dirV            25.832     40.099  218.374   0.644 0.520117
R_dirYN              85.370     30.184 2245.241   2.828 0.004721 **
sent_dirN:R_dirYN   -32.449     43.849 2247.805  -0.740 0.459369
sent_dirV:R_dirYN    -5.581     42.583 2251.501  -0.131 0.895739
---
Signif. codes:  0 ‘***’ 0.001 ‘**’ 0.01 ‘*’ 0.05 ‘.’ 0.1 ‘ ’ 1

> anova (LMM)
Type III Analysis of Variance Table with Satterthwaite's method
                 Sum Sq Mean Sq NumDF   DenDF F value    Pr(>F)
type            1057649 1057649     1  114.18  5.7911 0.0177118 *
block           2874648 2874648     1  114.42 15.7400 0.0001271 ***
group              8748    8748     1   25.93  0.0479 0.8284719
sent_dir        5784252 2892126     2  114.07 15.8357 8.528e-07 ***
R_dir           3063341 3063341     1 2243.23 16.7732 4.362e-05 ***
sent_dir:R_dir   112661   56331     2 2250.64  0.3084 0.7346263
---
Signif. codes:  0 ‘***’ 0.001 ‘**’ 0.01 ‘*’ 0.05 ‘.’ 0.1 ‘ ’ 1

> eta_sq(LMM, partial = TRUE)
             term partial.etasq
1            type         0.048
2           block         0.121
3           group         0.002
4        sent_dir         0.217
5           R_dir         0.007
6  sent_dir:R_dir         0.000
```

### Table 6 - Experiment 6

#### 6a - Exp 6 Outliers computed with the standard method

```
> data = read.csv2("exp6.csv", sep=";", header=T)
> tapply(data$RT, list(data$compatib),mean)
       0        1
1802.441 1795.042

> data_l$RT<-log(data$RT)

> moments::skewness(data$RT)
[1] 1.910228
> moments::kurtosis(data$RT)
[1] 9.428649

> LMM<-lmer(RT~type+compatib+block+group+sent_dir+R_dir+(1|subj_code)+(1|sent_code),data=data_l)
> summary(LMM)
Linear mixed model fit by REML. t-tests use Satterthwaite's method ['lmerModLmerTest']
Formula: RT ~ type + compatib + block + group + sent_dir + R_dir + (1 | subj_code) + (1 | sent_code)
   Data: data_l
REML criterion at convergence: 926
Scaled residuals:
    Min      1Q  Median      3Q     Max
-6.4006 -0.6835 -0.1198  0.5490  4.4472
Random effects:
 Groups   Name        Variance Std.Dev.
 sent_code (Intercept) 0.005092 0.07136
 subj_code (Intercept) 0.024926 0.15788
 Residual              0.074047 0.27212
Number of obs: 2817, groups:  sent_code, 80; subj_code, 42
Fixed effects:
              Estimate Std. Error         df t value Pr(>|t|)
(Intercept)  7.631e+00  4.876e-02  1.014e+02 156.509  < 2e-16 ***
typeC       -2.306e-02  1.902e-02  7.131e+01  -1.213   0.2292
compatib    -1.924e-03  1.029e-02  2.701e+03  -0.187   0.8517
block       -1.238e-01  1.900e-02  7.123e+01  -6.517  8.8e-09 ***
groupD      -6.555e-03  4.985e-02  4.015e+01  -0.131   0.8960
sent_dirV    3.811e-03  1.901e-02  7.132e+01   0.201   0.8416
R_dirYN      2.054e-02  1.028e-02  2.699e+03   1.997   0.0459 *
---
Signif. codes:  0 ‘***’ 0.001 ‘**’ 0.01 ‘*’ 0.05 ‘.’ 0.1 ‘ ’ 1

> anova(LMM)
Type III Analysis of Variance Table with Satterthwaite's method
          Sum Sq Mean Sq NumDF   DenDF F value    Pr(>F)
type      0.10890 0.10890     1   71.31  1.4707   0.22923
compatib  0.00259 0.00259     1 2700.85  0.0349   0.85174
block     3.14512 3.14512     1   71.23 42.4748 8.799e-09 ***
group     0.00128 0.00128     1   40.15  0.0173   0.89604
sent_dir  0.00298 0.00298     1   71.32  0.0402   0.84165
R_dir     0.29530 0.29530     1 2699.27  3.9881   0.04592 *
```

#### 6b - Exp 6 Outliers computed with the extended method

```
> data = read.csv2("exp6_e.csv", sep=";", header=T)
> moments::skewness(data$RT)
[1] 0.7196225
> moments::kurtosis(data$RT)
[1] 2.884276

> tapply(data$RT, list(data$block),mean)
       1        2
1781.840 1605.041

>  LMM<- lmer(RT~type+block+group+sent_dir+R_dir+sent_dir*R_dir+(1|subj_code)+(1|sent_code),data=data)
> summary (LMM)
Linear mixed model fit by REML. t-tests use Satterthwaite's method ['lmerModLmerTest']
Formula: RT ~ type + block + group + sent_dir + R_dir + sent_dir *      R_dir + (1 | subj_code) + (1 | sent_code)
   Data: data
REML criterion at convergence: 39786.2
Scaled residuals:
    Min      1Q  Median      3Q     Max
-2.5358 -0.7053 -0.1873  0.5382  3.7867
Random effects:
 Groups   Name        Variance Std.Dev.
 sent_code (Intercept)   9505    97.49
 subj_code (Intercept)  47738   218.49
 Residual              167366   409.10
Number of obs: 2667, groups:  sent_code, 80; subj_code, 42
Fixed effects:
                   Estimate Std. Error       df t value Pr(>|t|)
(Intercept)        1973.280     68.646  102.026  28.746  < 2e-16 ***
typeC               -38.591     27.054   71.381  -1.426    0.158
block              -177.221     27.022   71.268  -6.558  7.4e-09 ***
groupD               -9.198     69.367   39.826  -0.133    0.895
sent_dirV            15.132     31.292  127.758   0.484    0.630
R_dirYN              26.471     22.554 2545.925   1.174    0.241
sent_dirV:R_dirYN    25.347     31.815 2551.210   0.797    0.426
---
Signif. codes:  0 ‘***’ 0.001 ‘**’ 0.01 ‘*’ 0.05 ‘.’ 0.1 ‘ ’ 1

> anova(LMM)
Type III Analysis of Variance Table with Satterthwaite's method
                 Sum Sq Mean Sq NumDF   DenDF F value  Pr(>F)
type             340536  340536     1   71.38  2.0347 0.15811
block           7198635 7198635     1   71.27 43.0113 7.4e-09 ***
group              2943    2943     1   39.83  0.0176 0.89517
sent_dir         177047  177047     1   71.36  1.0578 0.30718
R_dir           1014832 1014832     1 2549.72  6.0635 0.01387 *
sent_dir:R_dir   106234  106234     1 2551.21  0.6347 0.42570
---
Signif. codes:  0 ‘***’ 0.001 ‘**’ 0.01 ‘*’ 0.05 ‘.’ 0.1 ‘ ’ 1

> eta_sq(LMM, partial = TRUE)
             term partial.etasq
1            type         0.028
2           block         0.376
3           group         0.000
4        sent_dir         0.015
5           R_dir         0.002
6  sent_dir:R_dir         0.000

> tapply(data$RT, list(data$R_dir),mean)
      YF       YN
1671.164 1713.465
```

#### 6c - Exp 6 Separate analysis for Block 1

```
> block1 <- data[ which(block==1),]
> LMM= lmer(RT ~ type + sent_dir + R_dir + sent_dir * R_dir + (1 |      subj_code) + (1 | sent_code) ,data=block1)
> summary(LMM)
Linear mixed model fit by REML. t-tests use Satterthwaite's method ['lmerModLmerTest']
Formula: RT ~ type + sent_dir + R_dir + sent_dir * R_dir + (1 | subj_code) + (1 | sent_code)
   Data: block1
REML criterion at convergence: 19633.8
Scaled residuals:
    Min      1Q  Median      3Q     Max
-2.2310 -0.7338 -0.1707  0.6200  3.3432
Random effects:
 Groups   Name        Variance Std.Dev.
 subj_code  (Intercept)  49686   222.90
 sent_code  (Intercept)   5486    74.07
 Residual               170878   413.37
Number of obs: 1314, groups:  subj_code, 42; sent_code, 40
Fixed effects:
                   Estimate Std. Error       df t value Pr(>|t|)
(Intercept)        1799.956     59.233   61.716  30.388   <2e-16 ***
typeC               -20.148     32.859   35.973  -0.613    0.544
sent_dirV            -7.063     40.182   80.109  -0.176    0.861
R_dirYN              12.480     76.244   47.927   0.164    0.871
sent_dirV:R_dirYN    21.199     45.852 1237.315   0.462    0.644
```

#### 6d - Exp 6 Separate analysis for Block 2

```
> block2 <- data[ which(block==2),]
> LMM= lmer(RT ~ type + sent_dir + R_dir + sent_dir * R_dir + (1 | subj_code) + (1 | sent_code) ,data=block2)
> summary(LMM)
Linear mixed model fit by REML. t-tests use Satterthwaite's method ['lmerModLmerTest']
Formula: RT ~ type + sent_dir + R_dir + sent_dir * R_dir + (1 | subj_code) + (1 | sent_code)
   Data: block2
REML criterion at convergence: 20145.7
Scaled residuals:
    Min      1Q  Median      3Q     Max
-2.1076 -0.6800 -0.1921  0.4852  4.0502
Random effects:
 Groups   Name        Variance Std.Dev.
 subj_code  (Intercept)  50269   224.2
 sent_code  (Intercept)  13109   114.5
 Residual               159228   399.0
Number of obs: 1353, groups:  subj_code, 42; sent_code, 40
Fixed effects:
                   Estimate Std. Error      df t value Pr(>|t|)
(Intercept)         1610.18      63.12   69.14  25.508   <2e-16 ***
typeC                -57.90      42.37   34.00  -1.367    0.181
sent_dirV             35.68      47.37   53.10   0.753    0.455
R_dirYN               27.01      75.89   47.40   0.356    0.724
sent_dirV:R_dirYN     33.69      43.66 1274.77   0.772    0.440
```

#### 6e - Exp 6 Analysis including no-transfer sentences

```
> data = read.csv2("exp6_n.csv", sep=";", header=T)
> tapply(data$RT, list(data$sent_dir),mean)
      A        N        T
1679.375 1816.451 1704.739
>  LMM<- lmer(RT~type+block+group+sent_dir+R_dir+sent_dir*R_dir+(1|subj_code)+(1|sent_code),data=data)
> summary (LMM)
Linear mixed model fit by REML. t-tests use Satterthwaite's method ['lmerModLmerTest']
Formula: RT ~ type + block + group + sent_dir + R_dir + sent_dir * R_dir + (1 | subj_code) + (1 | sent_code)
   Data: data
REML criterion at convergence: 57717.1
Scaled residuals:
    Min      1Q  Median      3Q     Max
-2.4658 -0.7016 -0.1655  0.5508  3.8163
Random effects:
 Groups   Name        Variance Std.Dev.
 sent_code (Intercept)  13319   115.4
 subj_code  (Intercept)  47045   216.9
 Residual             163400   404.2
Number of obs: 3875, groups:  sent_code, 120; subj_code, 42
Fixed effects:
                   Estimate Std. Error      df t value Pr(>|t|)
(Intercept)         1988.39      66.70  112.77  29.810  < 2e-16 ***
typeC                -62.08      24.85  107.82  -2.498    0.014 *
block               -177.69      24.84  107.77  -7.153 1.07e-10 ***
groupD               -15.00      68.26   39.94  -0.220    0.827
sent_dirN            160.03      34.49  177.57   4.640 6.76e-06 ***
sent_dirV             16.06      34.03  168.81   0.472    0.638
R_dirYN               26.85      22.28 3711.33   1.206    0.228
sent_dirN:R_dirYN    -16.41      32.30 3719.90  -0.508    0.611
sent_dirV:R_dirYN     25.10      31.42 3717.33   0.799    0.425
---
Signif. codes:  0 ‘***’ 0.001 ‘**’ 0.01 ‘*’ 0.05 ‘.’ 0.1 ‘ ’ 1

> anova(LMM)
Type III Analysis of Variance Table with Satterthwaite's method
                 Sum Sq Mean Sq NumDF  DenDF F value    Pr(>F)
type            1019410 1019410     1  107.8  6.2388   0.01401 *
block           8361545 8361545     1  107.8 51.1724 1.072e-10 ***
group              7894    7894     1   39.9  0.0483   0.82715
sent_dir        4545921 2272961     2  107.8 13.9104 4.223e-06 ***
R_dir            848736  848736     1 3719.2  5.1942   0.02272 *
sent_dir:R_dir   277780  138890     2 3720.5  0.8500   0.42750
---
Signif. codes:  0 ‘***’ 0.001 ‘**’ 0.01 ‘*’ 0.05 ‘.’ 0.1 ‘ ’ 1

> eta_sq(LMM, partial = TRUE)
             term partial.etasq
1            type         0.055
2           block         0.322
3           group         0.001
4        sent_dir         0.205
5           R_dir         0.001
6  sent_dir:R_dir         0.000

> tapply(data$RT, list(data$R_dir),mean)
      YF       YN
1714.544 1747.413

> tapply(data$RT, list(data$type),mean)
       A        C
1756.288 1706.699
```

### Table 7 – Comparison across experiments of sensibleness by sentence direction

| **Exp 1** | A | N | T | Total | |
| --- | --- | --- | --- | --- | --- |
| N | 1439,46 | 1481,34 | 1496,69 | 1472,51 | |
| S | 1408,28 | 1509,37 | 1479,13 | 1464,84 | |
| Total | 1423,77 | 1495,10 | 1487,99 | 1468,70 | |
| **Exp 2** | A | N | T | Total | |
| N | 1453,31 | 1542,96 | 1452,25 | 1482,50 | |
| S | 1442,84 | 1602,11 | 1445,62 | 1496,86 | |
| Total | 1448,13 | 1572,73 | 1448,91 | 1489,68 | |
| **Exp 3** | A | N | T | Total | |
| N | 1558,11 | 1643,78* | 1586,26 | 1595,58 | |
| S | 1567,68 | 1871,50 * | 1557,51 | 1665,58 | |
| Total | 1562,90 | 1759,08 | 1572,02 | 1630,63 | |
| **Exp 4** | A | N | T | Total | |
| N | 1662,47 | 1731,38* | 1681,16 | 1691,15 | |
| S | 1638,34 | 1805,99* | 1683,96 | 1707,41 | |
| Total | 1650,64 | 1767,72 | 1682,56 | 1699,16 | |
| **Exp 5** | A | N | T | Total |  |
| N | 1651,88 | 1747,32* | 1739,58 | 1712,59 |  |
| S | 1666,47 | 1822,73* | 1677,26 | 1719,28 |  |
| Total | 1659,15 | 1783,82 | 1708,53 | 1715,89 |  |
| **Exp 6** | A | N | T | Total |  |
| N | 1692,28 | 1732,59* | 1746,37 | 1723,67 |  |
| S | 1690,78 | 1822,07* | 1719,50 | 1742,53 |  |
| Total | 1691,53 | 1777,16 | 1732,87 | 1733,11 |  |

N= Nonsense, S = Sensible

A = Away, N= No-transfer, T= Towards

* significant difference, p < .05

### Comparison across experiments of sensibleness by sentence direction

### (procedure details)

#### Exp 1 (non sig.)

```
> data = read.csv2("exp1_nonsense.csv", sep=";", header=T)
> tapply(data$RT, list(data$sense, data$sent_dir),mean)
         A        N        T
N 1439.459 1481.336 1496.693
S 1408.280 1509.367 1479.132

> LMM<-lmer(RT~sense+sent_dir+sense*sent_dir+(1|subj_code)+(1|sent_code),data=data)
> summary(LMM)
Linear mixed model fit by REML
t-tests use  Satterthwaite approximations to degrees of freedom ['lmerMod']
Formula: RT ~ sense + sent_dir + sense * sent_dir + (1 | subj_code) +      (1 | sent_code)
   Data: data
REML criterion at convergence: 79188.4
Scaled residuals:
    Min      1Q  Median      3Q     Max
-2.3782 -0.6764 -0.1767  0.4810  4.0364

Random effects:
 Groups   Name        Variance Std.Dev.
 sent_code (Intercept)  19033   138.0
 subj_code (Intercept)  67094   259.0
 Residual              167957   409.8
Number of obs: 5302, groups:  sent_code, 240; subj_code, 25
Fixed effects:
                Estimate Std. Error      df t value Pr(>|t|)
(Intercept)       1451.55      57.88   36.24  25.080   <2e-16 ***
senseS             -39.64      36.46  230.01  -1.087    0.278
sent_dirN           40.33      36.48  230.55   1.106    0.270
sent_dirV           50.05      36.49  230.85   1.372    0.171
senseS:sent_dirN    79.28      51.64  231.37   1.535    0.126
senseS:sent_dirV    27.04      51.61  231.02   0.524    0.601
```

#### Exp 2 (non sig)

```
> data = read.csv2("exp2_nonsense.csv", sep=";", header=T)
> tapply(data$RT, list(data$sense, data$sent_dir),mean)
         A        N        T
N 1453.308 1542.964 1452.253
S 1442.841 1602.112 1445.618
> LMM<-lmer(RT~sense+sent_dir+sense*sent_dir+(1|subj_code)+(1|sent_code),data=data)
> summary(LMM)
Linear mixed model fit by REML
t-tests use  Satterthwaite approximations to degrees of freedom ['lmerMod']
Formula: RT ~ sense + sent_dir + sense * sent_dir + (1 | subj_code) +      (1 | sent_code)
   Data: data
REML criterion at convergence: 77905
Scaled residuals:
    Min      1Q  Median      3Q     Max
-2.5429 -0.6228 -0.1989  0.3858  5.1391
Random effects:
 Groups   Name        Variance Std.Dev.
 sent_code (Intercept)  25749   160.5
 subj_code (Intercept)  43826   209.3
 Residual              214098   462.7
Number of obs: 5133, groups:  sent_code, 240; subj_code, 24
Fixed effects:
                Estimate Std. Error       df t value Pr(>|t|)
(Intercept)      1454.602     52.103   47.464  27.918   <2e-16 ***
senseS            -12.550     42.230  231.065  -0.297   0.7666
sent_dirN          88.777     42.274  232.029   2.100   0.0368 *
sent_dirV           1.418     42.281  232.165   0.034   0.9733
senseS:sent_dirN   79.797     59.804  232.326   1.334   0.1834
senseS:sent_dirV    3.035     59.815  232.477   0.051   0.9596
```

#### EXP 3

```
> data = read.csv2("exp3_nonsense.csv", sep=";", header=T)
> tapply(data$RT, list(data$sense, data$sent_dir),mean)
         A        N        T
N 1558.111 1643.782 1586.257
S 1567.681 1871.498 1557.508

> LMM<-lmer(RT~sense+sense*sent_dir+(1|subj_code)+(1|sent_code),data=data)
> summary(LMM)
Linear mixed model fit by REML
t-tests use  Satterthwaite approximations to degrees of freedom ['lmerMod']
Formula: RT ~ sense + sense * sent_dir + (1 | subj_code) + (1 | sent_code)
   Data: data
REML criterion at convergence: 69557.4
Scaled residuals:
    Min      1Q  Median      3Q     Max
-2.2759 -0.4763 -0.1730  0.2164 15.5777
Random effects:
 Groups   Name        Variance Std.Dev.
 sent_code (Intercept)  55979   236.6
 subj_code (Intercept) 103046   321.0
 Residual              542131   736.3
Number of obs: 4320, groups:  sent_code, 240; subj_code, 20
Fixed effects:
                Estimate Std. Error       df t value Pr(>|t|)
(Intercept)      1562.492     85.475   35.919  18.280   <2e-16 ***
senseS              6.781     65.603  233.814   0.103   0.9178
sent_dirN          77.678     65.787  236.332   1.181   0.2389
sent_dirV          28.751     65.551  232.998   0.439   0.6614
senseS:sent_dirN  221.489     92.910  235.127   2.384   0.0179 *
senseS:sent_dirV  -44.293     92.769  233.647  -0.477   0.6335
---
Signif. codes:  0 ‘***’ 0.001 ‘**’ 0.01 ‘*’ 0.05 ‘.’ 0.1 ‘ ’ 1
```

#### EXP 4

```
> data = read.csv2("exp4_nonsense.csv", sep=";", header=T)
> tapply(data$RT, list(data$sense, data$sent_dir),mean)
         A        N        T
N 1662.465 1731.379 1681.160
S 1638.342 1805.988 1683.961

> LMM<-lmer(RT~sense+sent_dir+sense*sent_dir+(1|subj_code)+(1|sent_code),data=data)
> summary(LMM)
Linear mixed model fit by REML
t-tests use  Satterthwaite approximations to degrees of freedom ['lmerMod']
Formula: RT ~ sense + sent_dir + sense * sent_dir + (1 | subj_code) +      (1 | sent_code)
   Data: data
REML criterion at convergence: 61235.7
Scaled residuals:
    Min      1Q  Median      3Q     Max
-2.7022 -0.6860 -0.1585  0.5708  3.8232
Random effects:
 Groups   Name        Variance Std.Dev.
 sent_code (Intercept)  17335   131.7
 subj_code (Intercept)  65864   256.6
 Residual              173846   416.9
Number of obs: 4091, groups:  sent_code, 240; subj_code, 20
Fixed effects:
                Estimate Std. Error      df t value Pr(>|t|)
(Intercept)       1679.03      63.05   26.71  26.632   <2e-16 ***
senseS             -20.70      37.05  223.85  -0.559   0.5770
sent_dirN           78.36      37.07  224.25   2.114   0.0356 *
sent_dirV           23.95      36.97  221.91   0.648   0.5178
senseS:sent_dirN   107.63      52.67  228.23   2.043   0.0422 *
senseS:sent_dirV    25.31      52.36  223.26   0.483   0.6293
```

#### EXP 5

```
> data = read.csv2("exp5_nonsense.csv", sep=";", header=T)
> tapply(data$RT, list(data$sense, data$sent_dir),mean)
         A        N        T
N 1651.883 1747.320 1739.577
S 1666.473 1822.725 1677.256

> LMM<-lmer(RT~sense+sent_dir+sense*sent_dir+(1|subj_code)+(1|sent_code),data=data)
> summary(LMM)
Linear mixed model fit by REML
t-tests use  Satterthwaite approximations to degrees of freedom ['lmerMod']
Formula: RT ~ sense + sent_dir + sense * sent_dir + (1 | subj_code) +      (1 | sent_code)
   Data: data
REML criterion at convergence: 74884
Scaled residuals:
    Min      1Q  Median      3Q     Max
-2.5961 -0.7121 -0.1772  0.5809  3.9535
Random effects:
 Groups   Name        Variance Std.Dev.
 sent_code (Intercept)  15966   126.4
 subj_code (Intercept)  56399   237.5
 Residual              193739   440.2
Number of obs: 4969, groups:  sent_code, 240; subj_code, 28
Fixed effects:
                Estimate Std. Error      df t value Pr(>|t|)
(Intercept)       1659.64      51.43   44.08  32.267  < 2e-16 ***
senseS              10.63      35.52  227.01   0.299  0.76510
sent_dirN           97.25      35.60  228.98   2.732  0.00679 **
sent_dirV           87.14      35.50  226.34   2.455  0.01485 *
senseS:sent_dirN    78.01      50.56  232.17   1.543  0.12420
senseS:sent_dirV   -63.18      50.24  226.93  -1.257  0.20989
---
Signif. codes:  0 ‘***’ 0.001 ‘**’ 0.01 ‘*’ 0.05 ‘.’ 0.1 ‘ ’ 1

> anova(LMM)
Analysis of Variance Table of type III  with  Satterthwaite
approximation for degrees of freedom
               Sum Sq Mean Sq NumDF  DenDF F.value    Pr(>F)
sense          110687  110687     1 230.51  0.5713    0.4505
sent_dir       5684057 2842029     2 230.46 14.6694 1.008e-06 ***
sense:sent_dir 1514787  757394     2 230.38  3.9093    0.0214 *
```

#### EXP 6

```
> data = read.csv2("exp6_nonsense.csv", sep=";", header=T)
> tapply(data$RT, list(data$sense, data$sent_dir),mean)
         A        N        T
N 1692.285 1732.594 1746.366
S 1690.778 1822.067 1719.496

> LMM<-lmer(RT~sense+sent_dir+sense*sent_dir+(1|subj_code)+(1|sent_code),data=data)
> summary(LMM)
Linear mixed model fit by REML
t-tests use  Satterthwaite approximations to degrees of freedom ['lmerMod']
Formula: RT ~ sense + sent_dir + sense * sent_dir + (1 | subj_code) +      (1 | sent_code)
   Data: data
REML criterion at convergence: 123681.9
Scaled residuals:
    Min      1Q  Median      3Q     Max
-2.4103 -0.7097 -0.1860  0.5662  3.7144
Random effects:
 Groups   Name        Variance Std.Dev.
 sent_code (Intercept)  20057   141.6
 subj_code (Intercept)  42586   206.4
 Residual              175453   418.9
Number of obs: 8261, groups:  sent_code, 240; subj_code, 42
Fixed effects:
                Estimate Std. Error       df t value Pr(>|t|)
(Intercept)      1710.944     40.527   96.123  42.218   <2e-16 ***
senseS             -4.335     35.442  228.339  -0.122   0.9028
sent_dirN          44.036     35.546  230.934   1.239   0.2167
sent_dirV          53.568     35.429  228.043   1.512   0.1319
senseS:sent_dirN  105.053     50.284  231.151   2.089   0.0378 *
senseS:sent_dirV  -25.122     50.089  227.769  -0.502   0.6165
---
Signif. codes:  0 ‘***’ 0.001 ‘**’ 0.01 ‘*’ 0.05 ‘.’ 0.1 ‘ ’ 1

> anova(LMM)
Analysis of Variance Table of type III  with  Satterthwaite
approximation for degrees of freedom
               Sum Sq Mean Sq NumDF  DenDF F.value    Pr(>F)
sense          207840  207840     1 229.81  1.1846 0.2775635
direz_F       2606425 1303213     2 229.80  7.4277 0.0007485 ***
sense:direz_F 1322165  661083     2 229.82  3.7679 0.0245412 *
---
Signif. codes:  0 ‘***’ 0.001 ‘**’ 0.01 ‘*’ 0.05 ‘.’ 0.1 ‘ ’ 1
```

# Table 8 – Pooled analysis

```
> data = read.csv2("pooled.csv", sep=";", header=T)

> moments::skewness(data$RT)
[1] 0.6630489
> moments::kurtosis(data$RT)
[1] 2.889858
excess kurtosis = -.11

> LMM <-lmer(RT~type+block+group+sent_dir+R_dir+sent_dir*R_dir+(1|subj_code)+(1|sent_code)+(1|experim),data=data)
> summary(LMM)
Linear mixed model fit by REML. t-tests use Satterthwaite's method ['lmerModLmerTest']
Formula: RT ~ type + block + group + sent_dir + R_dir + sent_dir * R_dir + (1 | subj_code) + (1 | sent_code) + (1 | experim)
   Data: data

REML criterion at convergence: 380007.3

Scaled residuals:
    Min      1Q  Median      3Q     Max
-3.1753 -0.6859 -0.1836  0.5334  4.3862

Random effects:
 Groups    Name        Variance Std.Dev.
 sent_code (Intercept)  11680   108.1
 subj_code (Intercept)  48217   219.6
 experim   (Intercept)  16932   130.1
 Residual              166825   408.4
Number of obs: 25502, groups:  sent_code, 240; subj_code, 134; experim, 5

Fixed effects:
                    Estimate Std. Error         df t value Pr(>|t|)
(Intercept)        1851.9800    69.8011     6.6419  26.532 5.45e-08 ***
typeC               -40.7395    14.7550   237.0878  -2.761  0.00621 **
block              -160.3936    14.8735   228.6851 -10.784  < 2e-16 ***
groupB              -20.0178    38.3072   127.5295  -0.523  0.60219
sent_dirN           115.7921    19.2978   287.9529   6.000 5.90e-09 ***
sent_dirT            28.9534    19.2247   283.7378   1.506  0.13317
R_dirYN              -0.0662     8.7855 25128.4502  -0.008  0.99399
sent_dirN:R_dirYN     2.3195    12.6333 25131.0504   0.184  0.85433
sent_dirT:R_dirYN    12.5723    12.4080 25130.8582   1.013  0.31096
---
Signif. codes:  0 ‘***’ 0.001 ‘**’ 0.01 ‘*’ 0.05 ‘.’ 0.1 ‘ ’ 1

> anova(LMM)
Type III Analysis of Variance Table with Satterthwaite's method
                 Sum Sq  Mean Sq NumDF   DenDF  F value    Pr(>F)
type            1271781  1271781     1   237.1   7.6234  0.006212 **
block          19400418 19400418     1   228.7 116.2920 < 2.2e-16 ***
group             45555    45555     1   127.5   0.2731  0.602187
sent_dir        7217569  3608785     2   228.7  21.6322 2.488e-09 ***
R_dir            152181   152181     1 25132.4   0.9122  0.339535
sent_dir:R_dir   193130    96565     2 25131.8   0.5788  0.560556
---
Signif. codes:  0 ‘***’ 0.001 ‘**’ 0.01 ‘*’ 0.05 ‘.’ 0.1 ‘ ’ 1

> tapply(data$RT, list(data$block),mean)
       1        2
1701.195 1551.711

> tapply(data$RT, list(data$sent_dir),mean)
       A        N        T
1579.230 1685.420 1614.284

> tapply(data$RT, list(data$type),mean)
       A        C
1642.721 1607.945


> eta_sq(LMM, partial = TRUE)
            term partial.etasq
1           type         0.031
2          block         0.337
3          group         0.002
4       sent_dir         0.159
5          R_dir         0.000
6 sent_dir:R_dir         0.000
```

# Table 9 – Bayesian analysis

Bayesian ANOVA was conducted using JASP 0.14.1 software (JASP Team, 2020) and using JASP’s default priors (r scale = 0.5 for fixed effects and 1.0 for random effects).

Dependent variable = RT; Fixed factors = R_dir, sent_dir;
Random factors = subj_code, sent_code

**Experiment 2**

| **Model Comparison** | | | | | | | | | | | | | | | | | | | | | | | | | | | |
| --- | --- | --- | --- | --- | --- | --- | --- | --- | --- | --- | --- | --- | --- | --- | --- | --- | --- | --- | --- | --- | --- | --- | --- | --- | --- | --- | --- |
| **Models** | | | | | | | | | | | **P(M)** | | | | **P(M\|data)** | | | | **BF _M_** | | | | **BF _01_** | | **error %** | | |
| subj_code + sent_code | | | | | | | | | |  | 0.125 | | |  | 0.911 | | |  | 71.519 | | |  | 1.000 |  | |  |  |
| subj_code + sent_code + R_dir  ✻  sent_dir | | | | | | | | | |  | 0.125 | | |  | 0.089 | | |  | 0.685 | | |  | 10.217 |  | | 3.555 |  |
| subj_code | | | | | | | | | |  | 0.125 | | |  | 1.699e  -35 | | |  | 1.189e  -34 | | |  | 5.360e  +34 |  | | 2.966 |  |
| subj_code + R_dir  ✻  sent_dir | | | | | | | | | |  | 0.125 | | |  | 1.577e  -36 | | |  | 1.104e  -35 | | |  | 5.774e  +35 |  | | 3.511 |  |
| sent_code | | | | | | | | | |  | 0.125 | | |  | 9.755e -127 | | |  | 6.829e -126 | | |  | 9.337e +125 |  | | 4.755 |  |
| sent_code + R_dir  ✻  sent_dir | | | | | | | | | |  | 0.125 | | |  | 8.789e -128 | | |  | 6.152e -127 | | |  | 1.036e +127 |  | | 3.265 |  |
| Null model (incl. R_dir, sent_dir) | | | | | | | | | |  | 0.125 | | |  | 5.603e -149 | | |  | 3.922e -148 | | |  | 1.626e +148 |  | | 2.049 |  |
| R_dir  ✻  sent_dir | | | | | | | | | |  | 0.125 | | |  | 5.795e -150 | | |  | 4.056e -149 | | |  | 1.572e +149 |  | | 6.562 |  |
|  | | | | | | | | | | | | | | | | | | | | | | | | | | | |
| *Note.*  All models include R_dir, sent_dir | | | | | | | | | | | | | | | | | | | | | | | | | | | |
| **Analysis of Effects - RT** | | | | | | | | | | | | | | | | | | | | |  | | | | | | |
| **Effects** | | **P(incl)** | | | **P(excl)** | | | **P(incl\|data)** | | | | | **P(excl\|data)** | | | | **BF _excl_** | | | |  | | | | | | |
| subj_code |  | | 0.500 |  | | 0.500 |  | | 1.000 | | |  | 0.000 | | |  | 0.000 | | |  |  | | | | | | |
| sent_code |  | | 0.500 |  | | 0.500 |  | | 1.000 | | |  | 0.000 | | |  | 0.000 | | |  |  | | | | | | |
| R_dir  ✻  sent_dir |  | | 0.500 |  | | 0.500 |  | | 0.089 | | |  | 0.911 | | |  | 10.217 | | |  |  | | | | | | |
|  | | | | | | | | | | | | | | | | | | | | |  | | | | | | |

**Experiment 3**

| **Model Comparison** | | | | | | | | | | | | | | | | | | | | | | | | | | | |
| --- | --- | --- | --- | --- | --- | --- | --- | --- | --- | --- | --- | --- | --- | --- | --- | --- | --- | --- | --- | --- | --- | --- | --- | --- | --- | --- | --- |
| **Models** | | | | | | | | | | | **P(M)** | | | | **P(M\|data)** | | | | **BF _M_** | | | | **BF _01_** | | **error %** | | |
| subj_code + sent_code | | | | | | | | | |  | 0.125 | | |  | 0.942 | | |  | 113.427 | | |  | 1.000 |  | |  |  |
| subj_code + sent_code + R_dir  ✻  sent_dir | | | | | | | | | |  | 0.125 | | |  | 0.058 | | |  | 0.432 | | |  | 16.204 |  | | 7.607 |  |
| subj_code | | | | | | | | | |  | 0.125 | | |  | 3.444e  -28 | | |  | 2.411e  -27 | | |  | 2.735e  +27 |  | | 7.195 |  |
| subj_code + R_dir  ✻  sent_dir | | | | | | | | | |  | 0.125 | | |  | 2.099e  -29 | | |  | 1.469e  -28 | | |  | 4.487e  +28 |  | | 8.594 |  |
| sent_code | | | | | | | | | |  | 0.125 | | |  | 1.915e -155 | | |  | 1.341e -154 | | |  | 4.918e +154 |  | | 9.966 |  |
| sent_code + R_dir  ✻  sent_dir | | | | | | | | | |  | 0.125 | | |  | 1.165e -156 | | |  | 8.153e -156 | | |  | 8.087e +155 |  | | 8.130 |  |
| Null model (incl. R_dir, sent_dir) | | | | | | | | | |  | 0.125 | | |  | 5.092e -166 | | |  | 3.564e -165 | | |  | 1.850e +165 |  | | 5.415 |  |
| R_dir  ✻  sent_dir | | | | | | | | | |  | 0.125 | | |  | 2.908e -167 | | |  | 2.035e -166 | | |  | 3.239e +166 |  | | 7.192 |  |
|  | | | | | | | | | | | | | | | | | | | | | | | | | | | |
| *Note.*  All models include R_dir, sent_dir | | | | | | | | | | | | | | | | | | | | | | | | | | | |
| **Analysis of Effects - RT** | | | | | | | | | | | | | | | | | | | | |  | | | | | | |
| **Effects** | | **P(incl)** | | | **P(excl)** | | | **P(incl\|data)** | | | | | **P(excl\|data)** | | | | **BF _excl_** | | | |  | | | | | | |
| subj_code |  | | 0.500 |  | | 0.500 |  | | 1.000 | | |  | 0.000 | | |  | 0.000 | | |  |  | | | | | | |
| sent_code |  | | 0.500 |  | | 0.500 |  | | 1.000 | | |  | 0.000 | | |  | 0.000 | | |  |  | | | | | | |
| R_dir  ✻  sent_dir |  | | 0.500 |  | | 0.500 |  | | 0.058 | | |  | 0.942 | | |  | 16.204 | | |  |  | | | | | | |
|  | | | | | | | | | | | | | | | | | | | | |  | | | | | | |

**Experiment 4**

| **Model Comparison** | | | | | | | | | | | | | | | | | | | | | | | | | | | |
| --- | --- | --- | --- | --- | --- | --- | --- | --- | --- | --- | --- | --- | --- | --- | --- | --- | --- | --- | --- | --- | --- | --- | --- | --- | --- | --- | --- |
| **Models** | | | | | | | | | | | **P(M)** | | | | **P(M\|data)** | | | | **BF _M_** | | | | **BF _01_** | | **error %** | | |
| subj_code + sent_code | | | | | | | | | |  | 0.125 | | |  | 0.941 | | |  | 112.466 | | |  | 1.000 |  | |  |  |
| subj_code + sent_code + R_dir  ✻  sent_dir | | | | | | | | | |  | 0.125 | | |  | 0.059 | | |  | 0.436 | | |  | 16.067 |  | | 4.815 |  |
| subj_code | | | | | | | | | |  | 0.125 | | |  | 7.859e  -13 | | |  | 5.501e  -12 | | |  | 1.198e  +12 |  | | 4.541 |  |
| subj_code + R_dir  ✻  sent_dir | | | | | | | | | |  | 0.125 | | |  | 4.874e  -14 | | |  | 3.412e  -13 | | |  | 1.932e  +13 |  | | 4.312 |  |
| sent_code | | | | | | | | | |  | 0.125 | | |  | 1.005e -150 | | |  | 7.032e -150 | | |  | 9.371e +149 |  | | 4.993 |  |
| sent_code + R_dir  ✻  sent_dir | | | | | | | | | |  | 0.125 | | |  | 6.060e -152 | | |  | 4.242e -151 | | |  | 1.554e +151 |  | | 5.148 |  |
| Null model (incl. R_dir, sent_dir) | | | | | | | | | |  | 0.125 | | |  | 9.854e -154 | | |  | 6.898e -153 | | |  | 9.554e +152 |  | | 3.338 |  |
| R_dir  ✻  sent_dir | | | | | | | | | |  | 0.125 | | |  | 6.027e -155 | | |  | 4.219e -154 | | |  | 1.562e +154 |  | | 4.964 |  |
|  | | | | | | | | | | | | | | | | | | | | | | | | | | | |
| *Note.*  All models include R_dir, sent_dir | | | | | | | | | | | | | | | | | | | | | | | | | | | |
| **Analysis of Effects - RT** | | | | | | | | | | | | | | | | | | | | |  | | | | | | |
| **Effects** | | **P(incl)** | | | **P(excl)** | | | **P(incl\|data)** | | | | | **P(excl\|data)** | | | | **BF _excl_** | | | |  | | | | | | |
| subj_code |  | | 0.500 |  | | 0.500 |  | | 1.000 | | |  | 0.000 | | |  | 0.000 | | |  |  | | | | | | |
| sent_code |  | | 0.500 |  | | 0.500 |  | | 1.000 | | |  | 8.321e -13 | | |  | 8.321e -13 | | |  |  | | | | | | |
| R_dir  ✻  sent_dir |  | | 0.500 |  | | 0.500 |  | | 0.059 | | |  | 0.941 | | |  | 16.067 | | |  |  | | | | | | |
|  | | | | | | | | | | | | | | | | | | | | |  | | | | | | |

**Experiment 5**

| **Model Comparison** | | | | | | | | | | | | | | | | | | | | | | | | | | | |
| --- | --- | --- | --- | --- | --- | --- | --- | --- | --- | --- | --- | --- | --- | --- | --- | --- | --- | --- | --- | --- | --- | --- | --- | --- | --- | --- | --- |
| **Models** | | | | | | | | | | | **P(M)** | | | | **P(M\|data)** | | | | **BF _M_** | | | | **BF _01_** | | **error %** | | |
| subj_code + sent_code | | | | | | | | | |  | 0.125 | | |  | 0.945 | | |  | 121.074 | | |  | 1.000 |  | |  |  |
| subj_code + sent_code + R_dir  ✻  sent_dir | | | | | | | | | |  | 0.125 | | |  | 0.055 | | |  | 0.405 | | |  | 17.296 |  | | 6.667 |  |
| subj_code | | | | | | | | | |  | 0.125 | | |  | 2.819e  -17 | | |  | 1.973e  -16 | | |  | 3.354e  +16 |  | | 7.807 |  |
| subj_code + R_dir  ✻  sent_dir | | | | | | | | | |  | 0.125 | | |  | 1.446e  -18 | | |  | 1.012e  -17 | | |  | 6.536e  +17 |  | | 6.716 |  |
| sent_code | | | | | | | | | |  | 0.125 | | |  | 8.240e -146 | | |  | 5.768e -145 | | |  | 1.147e +145 |  | | 7.321 |  |
| sent_code + R_dir  ✻  sent_dir | | | | | | | | | |  | 0.125 | | |  | 4.417e -147 | | |  | 3.092e -146 | | |  | 2.140e +146 |  | | 7.472 |  |
| Null model (incl. R_dir, sent_dir) | | | | | | | | | |  | 0.125 | | |  | 1.064e -151 | | |  | 7.446e -151 | | |  | 8.887e +150 |  | | 4.656 |  |
| R_dir  ✻  sent_dir | | | | | | | | | |  | 0.125 | | |  | 5.741e -153 | | |  | 4.019e -152 | | |  | 1.647e +152 |  | | 6.432 |  |
|  | | | | | | | | | | | | | | | | | | | | | | | | | | | |
| *Note.*  All models include R_dir, sent_dir | | | | | | | | | | | | | | | | | | | | | | | | | | | |
| **Analysis of Effects - RT** | | | | | | | | | | | | | | | | | | | | |  | | | | | | |
| **Effects** | | **P(incl)** | | | **P(excl)** | | | **P(incl\|data)** | | | | | **P(excl\|data)** | | | | **BF _excl_** | | | |  | | | | | | |
| subj_code |  | | 0.500 |  | | 0.500 |  | | 1.000 | | |  | 0.000 | | |  | 0.000 | | |  |  | | | | | | |
| sent_code |  | | 0.500 |  | | 0.500 |  | | 1.000 | | |  | 0.000 | | |  | 0.000 | | |  |  | | | | | | |
| R_dir  ✻  sent_dir |  | | 0.500 |  | | 0.500 |  | | 0.055 | | |  | 0.945 | | |  | 17.296 | | |  |  | | | | | | |
|  | | | | | | | | | | | | | | | | | | | | |  | | | | | | |

**Experiment 6**

| **Model Comparison** | | | | | | | | | | | | | | | | | | | | | | | | | | | |
| --- | --- | --- | --- | --- | --- | --- | --- | --- | --- | --- | --- | --- | --- | --- | --- | --- | --- | --- | --- | --- | --- | --- | --- | --- | --- | --- | --- |
| **Models** | | | | | | | | | | | **P(M)** | | | | **P(M\|data)** | | | | **BF _M_** | | | | **BF _01_** | | **error %** | | |
| subj_code + sent_code | | | | | | | | | |  | 0.125 | | |  | 0.729 | | |  | 18.828 | | |  | 1.000 |  | |  |  |
| subj_code + sent_code + R_dir  ✻  sent_dir | | | | | | | | | |  | 0.125 | | |  | 0.271 | | |  | 2.603 | | |  | 2.690 |  | | 5.185 |  |
| subj_code | | | | | | | | | |  | 0.125 | | |  | 1.183e  -53 | | |  | 8.281e  -53 | | |  | 6.162e  +52 |  | | 4.737 |  |
| subj_code + R_dir  ✻  sent_dir | | | | | | | | | |  | 0.125 | | |  | 2.769e  -54 | | |  | 1.938e  -53 | | |  | 2.633e  +53 |  | | 5.025 |  |
| sent_code | | | | | | | | | |  | 0.125 | | |  | 7.667e -203 | | |  | 5.367e -202 | | |  | 9.508e +201 |  | | 5.062 |  |
| sent_code + R_dir  ✻  sent_dir | | | | | | | | | |  | 0.125 | | |  | 1.175e -203 | | |  | 8.224e -203 | | |  | 6.205e +202 |  | | 5.658 |  |
| Null model (incl. R_dir, sent_dir) | | | | | | | | | |  | 0.125 | | |  | 3.189e -231 | | |  | 2.232e -230 | | |  | 2.286e +230 |  | | 3.266 |  |
| R_dir  ✻  sent_dir | | | | | | | | | |  | 0.125 | | |  | 3.828e -232 | | |  | 2.679e -231 | | |  | 1.904e +231 |  | | 4.752 |  |
|  | | | | | | | | | | | | | | | | | | | | | | | | | | | |
| *Note.*  All models include R_dir, sent_dir | | | | | | | | | | | | | | | | | | | | | | | | | | | |
| **Analysis of Effects - RT** | | | | | | | | | | | | | | | | | | | | |  | | | | | | |
| **Effects** | | **P(incl)** | | | **P(excl)** | | | **P(incl\|data)** | | | | | **P(excl\|data)** | | | | **BF _excl_** | | | |  | | | | | | |
| subj_code |  | | 0.500 |  | | 0.500 |  | | 1.000 | | |  | 8.882e -15 | | |  | 8.882e -15 | | |  |  | | | | | | |
| sent_code |  | | 0.500 |  | | 0.500 |  | | 1.000 | | |  | 8.882e -15 | | |  | 8.882e -15 | | |  |  | | | | | | |
| R_dir  ✻  sent_dir |  | | 0.500 |  | | 0.500 |  | | 0.271 | | |  | 0.729 | | |  | 2.690 | | |  |  | | | | | | |
|  | | | | | | | | | | | | | | | | | | | | |  | | | | | | |

# Full list of sentences

| **1** | Tu corteggi il tempo ad Anna (You woo Anna time) | nonsense | abstract | away |
| --- | --- | --- | --- | --- |
| **2** | Tu scali il giudizio a Daria (You step up to Daria's judgment) | nonsense | abstract | away |
| **3** | Tu guidi il premio a Gaetano (You guide the prize to Gaetano) | nonsense | abstract | away |
| **4** | Tu sali la democrazia a Lino (You climb democracy to Lino) | nonsense | abstract | away |
| **5** | Tu parli il seminario a Vito (You talk to Vito about the seminar) | nonsense | abstract | away |
| **6** | Tu corteggi il tempo con Ida (You woo time with Ida) | nonsense | abstract | no-transfer |
| **7** | Ugo scala il giudizio con te (Ugo climbs the judgment with you) | nonsense | abstract | no-transfer |
| **8** | Adele guida il premio con te (Adele drives the prize with you) | nonsense | abstract | no-transfer |
| **9** | Eva sala la democrazia con te (Eve climbs democracy with you) | nonsense | abstract | no-transfer |
| **10** | Tu parli il seminario con Ada (You talk about the seminar with Ada) | nonsense | abstract | no-transfer |
| **11** | Carmen ti corteggia il tempo (Carmen woos you time) | nonsense | abstract | toward |
| **12** | Marcello ti scala il giudizio (Marcello scales your judgment) | nonsense | abstract | toward |
| **13** | Alessandro ti guida il premio (Alessandro guides you the prize) | nonsense | abstract | toward |
| **14** | Susanna ti sala la democrazia (Susanna gives you democracy) | nonsense | abstract | toward |
| **15** | Tommaso ti parla il seminario (Thomas talks to you about the seminary) | nonsense | abstract | toward |
| **16** | Tu gestisci l’albero a Adele (You manage the tree in Adele) | nonsense | concrete | away |
| **17** | Tu urli la foglia a Raffaele (You scream the leaf at Raffaele) | nonsense | concrete | away |
| **18** | Tu ascolti il terreno a Lidia (You listen to the ground to Lydia) | nonsense | concrete | away |
| **19** | Tu canti la piramide a Marta (You sing the pyramid to Martha) | nonsense | concrete | away |
| **20** | Tu scrivi a Sandra il geranio (You write to Sandra the geranium) | nonsense | concrete | away |
| **21** | Luca gestisce l'albero con te (Luca manages the tree with you) | nonsense | concrete | no-transfer |
| **22** | Tu urli la foglia con Daniela (You scream the leaf with Daniela) | nonsense | concrete | no-transfer |
| **23** | Tu ascolti il terreno con Ida (You listen to the ground with Ida) | nonsense | concrete | no-transfer |
| **24** | Tu canti la piramide con Nino (You sing the pyramid with Nino) | nonsense | concrete | no-transfer |
| **25** | Enzo scrive il geranio con te (Enzo writes the geranium with you) | nonsense | concrete | no-transfer |
| **26** | Valerio ti gestisce l’albero (Valerio manages the tree to you) | nonsense | concrete | toward |
| **27** | Alessandro ti urla la foglia (Alessandro yells at you the leaf) | nonsense | concrete | toward |
| **28** | Gaetano ti ascolta il terreno (Gaetano listens to the ground) | nonsense | concrete | toward |
| **29** | Giorgio ti canta la piramide (Giorgio sings the pyramid to you) | nonsense | concrete | toward |
| **30** | Claudio ti scrive il geranio (Claudio writes you the geranium) | nonsense | concrete | toward |
| **31** | Tu comunichi a Daria l’avviso (You communicate the notice to Daria) | sensible | abstract | away |
| **32** | Tu racconti a Vito una storia (You tell Vito a story) | sensible | abstract | away |
| **33** | Tu racconti un fatto a Enrico (You tell Enrico a fact) | sensible | abstract | away |
| **34** | Tu deleghi l'incarico a Gino (You delegate the task to Gino) | sensible | abstract | away |
| **35** | Tu dedichi del tempo a Luigi (You spend time with Luigi) | sensible | abstract | away |
| **36** | Anna esamina con te l’avviso (Anna examines the notice with you) | sensible | abstract | no-transfer |
| **37** | Ivano legge una storia con te (Ivano reads a story with you) | sensible | abstract | no-transfer |
| **38** | Lucia discute un fatto con te (Lucia discusses a fact with you) | sensible | abstract | no-transfer |
| **39** | Tu e Edo esaminate l'incarico (You and Edo review the assignment) | sensible | abstract | no-transfer |
| **40** | Tu e Matteo passate del tempo (You and Matteo spend time) | sensible | abstract | no-transfer |
| **41** | Lauretta ti comunica l'avviso (Lauretta gives you the notice) | sensible | abstract | toward |
| **42** | Debora ti racconta una storia (Debora tells you a story) | sensible | abstract | toward |
| **43** | Rebecca ti racconta un fatto (Rebecca tells you a fact) | sensible | abstract | toward |
| **44** | Massimo ti delega l'incarico (Massimo delegates the task to you) | sensible | abstract | toward |
| **45** | Eva ti dedica un po’ di tempo (Eva dedicates some time to you) | sensible | abstract | toward |
| **46** | Tu dai le carte a Alessandro (You give the cards to Alessandro) | sensible | concrete | away |
| **47** | Tu porgi a Toni la fotografia (You hand Toni the photograph) | sensible | concrete | away |
| **48** | Tu servi la trippa a Adriano (You serve the tripe to Adriano) | sensible | concrete | away |
| **49** | Tu passi a Pietro la pittura (You pass the painting to Pietro) | sensible | concrete | away |
| **50** | Tu porgi i funghi a Raffaele (You hand the mushrooms to Raffaele) | sensible | concrete | away |
| **51** | Tu guardi le carte con Carla (You look at the cards with Carla) | sensible | concrete | no-transfer |
| **52** | Tu e Gio discutete della foto (You and Gio discuss the photo) | sensible | concrete | no-transfer |
| **53** | Tu prepari la trippa con Sara (You make tripe with Sara) | sensible | concrete | no-transfer |
| **54** | Tu mescoli la pittura con Lia (You mix paint with Lia) | sensible | concrete | no-transfer |
| **55** | Tu cuoci i funghi con Sandra (You cook the mushrooms with Sandra) | sensible | concrete | no-transfer |
| **56** | Alessandro ti dà le cartelle (Alessandro gives you the folders) | sensible | concrete | toward |
| **57** | Angela ti porge la fotografia (Angela hands you the photograph) | sensible | concrete | toward |
| **58** | Gianfranco ti serve la trippa (Gianfranco serves you the tripe) | sensible | concrete | toward |
| **59** | Francesco ti passa la pittura (Francesco hands you the painting) | sensible | concrete | toward |
| **60** | Gianfranco ti porge i funghi (Gianfranco hands you the mushrooms) | sensible | concrete | toward |
| **61** | Tu esplori l’ovazione a Marco (You explore the ovation to Marco) | nonsense | abstract | away |
| **62** | Tu presiedi l'idea a Lorenzo (You preside over the idea from Lorenzo) | nonsense | abstract | away |
| **63** | Tu accarezzi il canto a Giada (You caress the song to Giada) | nonsense | abstract | away |
| **64** | Tu profumi il diritto a Ciro (You perfume the right to Ciro) | nonsense | abstract | away |
| **65** | Tu nuoti la cerimonia a Anna (You swim the ceremony to Anna) | nonsense | abstract | away |
| **66** | Ale esplora l’ovazione con te (Ale explores ovation with you) | nonsense | abstract | no-transfer |
| **67** | Tu presiedi l'idea con Daria (You chair the idea with Daria) | nonsense | abstract | no-transfer |
| **68** | Tu accarezzi il canto con Ivo (You caress the song with Ivo) | nonsense | abstract | no-transfer |
| **69** | Tu profumi il diritto con Ada (You perfume the right with Ada) | nonsense | abstract | no-transfer |
| **70** | Ugo nuota la cerimonia con te (Ugo swims the ceremony with you) | nonsense | abstract | no-transfer |
| **71** | Carmen ti esplora l’ovazione (Carmen explores the ovation for you) | nonsense | abstract | toward |
| **72** | Gianfranco ti presiede l'idea (Gianfranco presides over the idea) | nonsense | abstract | toward |
| **73** | Ginevra ti accarezza il canto (Geneva caresses your song) | nonsense | abstract | toward |
| **74** | Marina ti profuma il diritto (Marina perfumes you the right) | nonsense | abstract | toward |
| **75** | Rebecca ti nuota la cerimonia (Rebecca swims the ceremony) | nonsense | abstract | toward |
| **76** | Tu versi il cavallo ad Ambra (You give the horse to Amber) | nonsense | concrete | away |
| **77** | Tu confermi la penna a Davide (You confirm the pen to David) | nonsense | concrete | away |
| **78** | Tu pensi a Giorgio la pallina (You think of Giorgio the ball) | nonsense | concrete | away |
| **79** | Tu confessi il piatto a Mara (You confess the dish to Mara) | nonsense | concrete | away |
| **80** | Tu fumi l’orecchino a Susanna (You smoke Susanna's earring) | nonsense | concrete | away |
| **81** | Alex versa il cavallo con te (Alex pours the horse with you) | nonsense | concrete | no-transfer |
| **82** | Tu confermi la penna con Alda (You confirm the pen with Alda) | nonsense | concrete | no-transfer |
| **83** | Lino pensa la pallina con te (Lino thinks the ball with you) | nonsense | concrete | no-transfer |
| **84** | Tu confessi il piatto con Ale (You confess the dish with Ale) | nonsense | concrete | no-transfer |
| **85** | Tu fumi l’orecchino con Susi (You smoke the earring with Susi) | nonsense | concrete | no-transfer |
| **86** | Marcella ti versa il cavallo (Marcella pours you the horse) | nonsense | concrete | toward |
| **87** | Valeria ti conferma la penna (Valeria confirms the pen) | nonsense | concrete | toward |
| **88** | Isabella ti pensa la pallina (Isabella thinks of the ball) | nonsense | concrete | toward |
| **89** | Angela ti confessa il piatto (Angela confesses the dish to you) | nonsense | concrete | toward |
| **90** | Pierpaolo ti fuma l’orecchino (Pierpaolo smokes your earring) | nonsense | concrete | toward |
| **91** | Tu dai ad Adriano un compito (You give Adriano a task) | sensible | abstract | away |
| **92** | Tu confidi un segreto a Diego (You are sharing a secret with Diego) | sensible | abstract | away |
| **93** | Tu fai una critica a Filippo (You make a criticism of Filippo) | sensible | abstract | away |
| **94** | Tu esprimi la tua stima a Lea (You express your esteem to Lea) | sensible | abstract | away |
| **95** | Tu porgi a Adele il cappello (You hand Adele your hat) | sensible | abstract | away |
| **96** | Dino pensa al compito con te (Dino thinks about the task with you) | sensible | abstract | no-transfer |
| **97** | Tu e Giorgia avete un segreto (You and Giorgia have a secret) | sensible | abstract | no-transfer |
| **98** | Lia pensa una critica con te (Lia thinks a criticism with you) | sensible | abstract | no-transfer |
| **99** | Lea parla con te della stima (Lea talks to you about esteem) | sensible | abstract | no-transfer |
| **100** | Tu e Eva comprate il cappello (You and Eva buy the hat) | sensible | abstract | no-transfer |
| **101** | Piergiorgio ti dà un compito (Piergiorgio gives you a task) | sensible | abstract | toward |
| **102** | Debora ti confida un segreto (Debora tells you a secret) | sensible | abstract | toward |
| **103** | Gianfranco ti fa una critica (Gianfranco makes you a criticism) | sensible | abstract | toward |
| **104** | Lidia ti esprime la sua stima (Lidia expresses her esteem) | sensible | abstract | toward |
| **105** | Nino ti fornisce il cappello (Nino gives you the hat) | sensible | abstract | toward |
| **106** | Tu dai le melanzane a Silvia (You give the aubergines to Silvia) | sensible | concrete | away |
| **107** | Tu offri il cucchiaio a Carla (You offer the spoon to Carla) | sensible | concrete | away |
| **108** | Tu dai il salmone a Francesca (You give the salmon to Francesca) | sensible | concrete | away |
| **109** | Tu fornisci a Ivo un articolo (You provide Ivo with an article) | sensible | concrete | away |
| **110** | Tu passi le uova a Simonetta (You pass the eggs to Simonetta) | sensible | concrete | away |
| **111** | Tu mangi le melanzane con Ale (You eat eggplant with Ale) | sensible | concrete | no-transfer |
| **112** | Tu lavi il cucchiaio con Ada (You wash the spoon with Ada) | sensible | concrete | no-transfer |
| **113** | Tu e Emma mangiate il salmone (You and Emma eat salmon) | sensible | concrete | no-transfer |
| **114** | Tu e Ida guardate un articolo (You and Ida look at an article) | sensible | concrete | no-transfer |
| **115** | Tu e Simona comprate le uova (You and Simona buy the eggs) | sensible | concrete | no-transfer |
| **116** | Donatella ti dà le melanzane (Donatella gives you the aubergines) | sensible | concrete | toward |
| **117** | Daniela ti offre il cucchiaio (Daniela offers you the spoon) | sensible | concrete | toward |
| **118** | Piergiorgio ti dà il salmone (Piergiorgio gives you the salmon) | sensible | concrete | toward |
| **119** | Luca ti fornisce un articolo (Luca gives you an article) | sensible | concrete | toward |
| **120** | Massimiliano ti passa le uova (Massimiliano passes you the eggs) | sensible | concrete | toward |
| **121** | Tu cuoci la correttezza a Lia (You cook fairness to Lia) | nonsense | abstract | away |
| **122** | Tu asciughi il film a Teresa (You dry Teresa's film) | nonsense | abstract | away |
| **123** | Tu pulisci la funzione a Ivo (You clean up the function in Ivo) | nonsense | abstract | away |
| **124** | Tu profumi l’analisi a Maria (You perfume Maria's analysis) | nonsense | abstract | away |
| **125** | Tu traballi un’idea a Teresa (You give Teresa an idea) | nonsense | abstract | away |
| **126** | Tu cuoci l'onestà con Claudia (You cook honesty with Claudia) | nonsense | abstract | no-transfer |
| **127** | Laura asciuga il film con te (Laura wipes the film with you) | nonsense | abstract | no-transfer |
| **128** | Tu mangi la funzione con Ivo (You eat function with Ivo) | nonsense | abstract | no-transfer |
| **129** | Tea profuma l’analisi con te (Tea perfumes analysis with you) | nonsense | abstract | no-transfer |
| **130** | Tu traballi un’idea con Tina (You have an idea with Tina) | nonsense | abstract | no-transfer |
| **131** | Mina ti cuoce la correttezza (Mina cooks you fairness) | nonsense | abstract | toward |
| **132** | Elisabetta ti asciuga il film (Elisabetta dries the film to you) | nonsense | abstract | toward |
| **133** | Luigi ti pulisce la funzione (Luigi cleans your function) | nonsense | abstract | toward |
| **134** | Mariella ti profuma l’analisi (Mariella perfumes analysis to you) | nonsense | abstract | toward |
| **135** | Eleonora ti traballa un’idea (Eleonora gives you an idea) | nonsense | abstract | toward |
| **136** | Tu pensi il tiro ad Antonino (You think the shot at Antonino) | nonsense | concrete | away |
| **137** | Tu impasti una bici a Fausto (You knead a bike to Fausto) | nonsense | concrete | away |
| **138** | Tu giochi il radiatore a Eva (You play the radiator to Eva) | nonsense | concrete | away |
| **139** | Tu canti le cartelle a Pietro (You sing the folders to Pietro) | nonsense | concrete | away |
| **140** | Tu dormi il treno a Emanuele (You sleep Emanuele's train) | nonsense | concrete | away |
| **141** | Antonio pensa il tiro con te (Antonio thinks the shot with you) | nonsense | concrete | no-transfer |
| **142** | Tu impasti una bici con Elisa (You knead a bike with Elisa) | nonsense | concrete | no-transfer |
| **143** | Edo gioca il radiatore con te (Edo plays the radiator with you) | nonsense | concrete | no-transfer |
| **144** | Pietro canta le carte con te (Pietro sings the cards with you) | nonsense | concrete | no-transfer |
| **145** | Tommaso dorme il treno con te (Tommaso sleeps the train with you) | nonsense | concrete | no-transfer |
| **146** | Massimiliano ti pensa il tiro (Massimiliano takes care of the shot) | nonsense | concrete | toward |
| **147** | Eleonora ti impasta una bici (Eleonora kneads a bike to you) | nonsense | concrete | toward |
| **148** | Iacopo ti gioca il radiatore (Iacopo plays the radiator to you) | nonsense | concrete | toward |
| **149** | Alessio ti canta le cartelle (Alessio sings you the folders) | nonsense | concrete | toward |
| **150** | Alessandro ti dorme il treno (Alessandro sleeps the train) | nonsense | concrete | toward |
| **151** | Tu comunichi a Edo la notizia (You give Edo the news) | sensible | abstract | away |
| **152** | Tu dai a Enrico un consiglio (You give Enrico some advice) | sensible | abstract | away |
| **153** | Tu rivolgi un reclamo a Susi (You make a complaint to Susi) | sensible | abstract | away |
| **154** | Tu esprimi la tua lode a Isa (You express your praise to Isa) | sensible | abstract | away |
| **155** | Tu comunichi gli ordini a Iva (You communicate the orders to Iva) | sensible | abstract | away |
| **156** | Tu e Linda sentite la notizia (You and Linda hear the news) | sensible | abstract | no-transfer |
| **157** | Ugo pensa con te un consiglio (Ugo thinks some advice with you) | sensible | abstract | no-transfer |
| **158** | Tu e Ale ascoltate un reclamo (You and Ale listen to a complaint) | sensible | abstract | no-transfer |
| **159** | Lucia parla con te delle lodi (Lucia talks to you about praise) | sensible | abstract | no-transfer |
| **160** | Tu e Tea rifiutate gli ordini (You and Tea refuse orders) | sensible | abstract | no-transfer |
| **161** | Marco ti comunica la notizia (Marco gives you the news) | sensible | abstract | toward |
| **162** | Marcellino ti dà un consiglio (Marcellin gives you some advice) | sensible | abstract | toward |
| **163** | Franco ti rivolge un reclamo (Franco makes a complaint to you) | sensible | abstract | toward |
| **164** | Lucia ti esprime la sua lode (Lucia expresses her praise to you) | sensible | abstract | toward |
| **165** | Sara ti impartisce gli ordini (Sara gives you the orders) | sensible | abstract | toward |
| **166** | Tu passi ad Andrea il foglio (You pass the sheet to Andrea) | sensible | concrete | away |
| **167** | Tu servi gli asparagi a Carla (You serve the asparagus to Carla) | sensible | concrete | away |
| **168** | Tu fornisci a Gigi la chiave (You give Gigi the key) | sensible | concrete | away |
| **169** | Tu tiri la pallina a Marcello (You throw the ball at Marcello) | sensible | concrete | away |
| **170** | Tu porgi a Marcello la tazza (You hand the cup to Marcello) | sensible | concrete | away |
| **171** | Tu guardi il foglio con Ambra (You look at the paper with Amber) | sensible | concrete | no-transfer |
| **172** | Tu e Edo assaggiate asparagi (You and Edo taste asparagus) | sensible | concrete | no-transfer |
| **173** | Tu esamini la chiave con Gigi (You examine the key with Gigi) | sensible | concrete | no-transfer |
| **174** | Tu e Mari guardate la pallina (You and Mari look at the ball) | sensible | concrete | no-transfer |
| **175** | Tu e Tonino annusate la tazza (You and Tonino smell the cup) | sensible | concrete | no-transfer |
| **176** | Gianfranco ti passa il foglio (Gianfranco hands you the sheet) | sensible | concrete | toward |
| **177** | Daria ti fa mangiare asparagi (Daria makes you eat asparagus) | sensible | concrete | toward |
| **178** | Antonio ti fornisce la chiave (Antonio gives you the key) | sensible | concrete | toward |
| **179** | Lia ti fa rotolare la pallina (Lia makes you roll the ball) | sensible | concrete | toward |
| **180** | Gianfilippo ti porge la tazza (Gianfilippo hands you the cup) | sensible | concrete | toward |
| **181** | Tu imbottigli la pace a Maria (You bottle up peace to Mary) | nonsense | abstract | away |
| **182** | Tu annoi il numero a Filippo (You bore Philip's number) | nonsense | abstract | away |
| **183** | Tu stiri la teoria a Isabella (You iron the theory out of Isabella) | nonsense | abstract | away |
| **184** | Tu concimi a Fausto la classe (You fertilize Fausto's class) | nonsense | abstract | away |
| **185** | Tu cammini la cura a Silvana (You walk the cure to Silvana) | nonsense | abstract | away |
| **186** | Tu imbottigli la pace con Ugo (You bottle up peace with Ugo) | nonsense | abstract | no-transfer |
| **187** | Tu annoi il numero con Pippo (You bore the number with Pippo) | nonsense | abstract | no-transfer |
| **188** | Linda stira la teoria con te (Linda irones theory with you) | nonsense | abstract | no-transfer |
| **189** | Ale concima la classe con te (Ale fertilizes the class with you) | nonsense | abstract | no-transfer |
| **190** | Adele cammina la cura con te (Adele walks the cure with you) | nonsense | abstract | no-transfer |
| **191** | Cinzia ti imbottiglia la pace (Cinzia bottles your peace) | nonsense | abstract | toward |
| **192** | Eleonora ti annoia il numero (Eleonora bores you the number) | nonsense | abstract | toward |
| **193** | Massimino ti stira la teoria (Massimino irones the theory) | nonsense | abstract | toward |
| **194** | Adriano ti concima la classe (Adriano fertilizes your class) | nonsense | abstract | toward |
| **195** | Francesca ti cammina la cura (Francesca walks you the cure) | nonsense | abstract | toward |
| **196** | Tu fondi il tavolo a Claudio (You found Claudio's table) | nonsense | concrete | away |
| **197** | Tu fingi il cemento a Fabiana (You fake the cement to Fabiana) | nonsense | concrete | away |
| **198** | Tu bevi la palla a Massimino (You drink Massimino's ball) | nonsense | concrete | away |
| **199** | Tu semini le trombe a Andrea (You sow the trumpets to Andrea) | nonsense | concrete | away |
| **200** | Tu bevi la stanza a Isabella (You drink Isabella's room) | nonsense | concrete | away |
| **201** | Tu fondi il tavolo con Marco (You found the table with Marco) | nonsense | concrete | no-transfer |
| **202** | Fabio finge il cemento con te (Fabio pretends concrete with you) | nonsense | concrete | no-transfer |
| **203** | Tu bevi la palla con Giorgio (You drink the ball with Giorgio) | nonsense | concrete | no-transfer |
| **204** | Renzo semina le trombe con te (Renzo sows the trumpets with you) | nonsense | concrete | no-transfer |
| **205** | Tu bevi la stanza con Valerio (You drink the room with Valerio) | nonsense | concrete | no-transfer |
| **206** | Simonetta ti fonde il tavolo (Simonetta melts your table) | nonsense | concrete | toward |
| **207** | Raffaella ti finge il cemento (Raffaella is pretending to be concrete) | nonsense | concrete | toward |
| **208** | Piergiorgio ti beve la palla (Piergiorgio drinks the ball) | nonsense | concrete | toward |
| **209** | Robertino ti semina le trombe (Robertino sows the trumpets) | nonsense | concrete | toward |
| **210** | Alessandra ti beve la stanza (Alessandra drinks your room) | nonsense | concrete | toward |
| **211** | Tu esponi l’argomento ad Eva (You explain the topic to Eve) | sensible | abstract | away |
| **212** | Tu trasmetti la nota a Eliana (You pass the note to Eliana) | sensible | abstract | away |
| **213** | Tu dedichi la canzone a Gino (You dedicate the song to Gino) | sensible | abstract | away |
| **214** | Tu suggerisci un’idea a Luigi (You suggest an idea to Luigi) | sensible | abstract | away |
| **215** | Tu dedichi una poesia a Sara (You dedicate a poem to Sara) | sensible | abstract | away |
| **216** | Tu e Isa esponete l’argomento (You and Isa explain the topic) | sensible | abstract | no-transfer |
| **217** | Tu e Fabio abbozzate la nota (You and Fabio sketch the note) | sensible | abstract | no-transfer |
| **218** | Tu e Lea ricordate la canzone (You and Lea remember the song) | sensible | abstract | no-transfer |
| **219** | Luigi discute un’idea con te (Luigi discusses an idea with you) | sensible | abstract | no-transfer |
| **220** | Susi legge una poesia con te (Susi reads a poem with you) | sensible | abstract | no-transfer |
| **221** | Massimo ti espone l’argomento (Massimo explains the topic to you) | sensible | abstract | toward |
| **222** | Luigi ti parla del messaggio (Luigi tells you about the message) | sensible | abstract | toward |
| **223** | Giada dedica la canzone a te (Giada dedicates the song to you) | sensible | abstract | toward |
| **224** | Tonino ti suggerisce un’idea (Tonino suggests you an idea) | sensible | abstract | toward |
| **225** | Silvia dedica una poesia a te (Silvia dedicates a poem to you) | sensible | abstract | toward |
| **226** | Tu passi il formaggio ad Anna (You pass the cheese to Anna) | sensible | concrete | away |
| **227** | Tu offri le carote a Claudio (You offer the carrots to Claudio) | sensible | concrete | away |
| **228** | Tu servi la frittata a Giuse (You serve the omelette to Giuse) | sensible | concrete | away |
| **229** | Tu lanci la palla a Isabella (You throw the ball to Isabella) | sensible | concrete | away |
| **230** | Tu offri il cioccolato a Ugo (You offer the chocolate to Ugo) | sensible | concrete | away |
| **231** | Tu gusti il formaggio con Ada (You taste cheese with Ada) | sensible | concrete | no-transfer |
| **232** | Tu e Diana mangiate le carote (You and Diana eat carrots) | sensible | concrete | no-transfer |
| **233** | Tu e Gio guardate la frittata (You and Gio look at the omelette) | sensible | concrete | no-transfer |
| **234** | Tu e Paolo guardate la palla (You and Paolo look at the ball) | sensible | concrete | no-transfer |
| **235** | Tu usi il cioccolato con Eva (You use chocolate with Eva) | sensible | concrete | no-transfer |
| **236** | Andrea ti passa il formaggio (Andrea hands you the cheese) | sensible | concrete | toward |
| **237** | Francesca ti offre le carote (Francesca offers you the carrots) | sensible | concrete | toward |
| **238** | Giuseppe ti serve la frittata (Giuseppe serves you the omelette) | sensible | concrete | toward |
| **239** | Raffaella ti lancia la palla (Raffaella throws the ball at you) | sensible | concrete | toward |
| **240** | Elisa ti offre il cioccolato (Elisa offers you the chocolate) | sensible | concrete | toward |

**Manuscript contribution to the field**

Briefly summarize in 200 words your manuscript’s contribution to, and position in, the existing literature of your field. This should be written avoiding any technical language or non-standard acronyms. The aim should be to convey the meaning and importance of this research to a non-expert. For examples, please see [author guidelines](http://home.frontiersin.org/about/author-guidelines#ContributionStatement). This statement is for editorial purposes and should not be included in your manuscript files, and will be removed if present.

According to a well-established paradigm, called “Action-sentence compatibility effect”, when we understand sentences that describe actions we reactivate, or “internally simulate”, sensory or motor past experiences. This hypothesis has been supported by classical experiments where participants were asked to judge the sensibleness of action-related sentences, by performing actions compatible or not compatible with the actions described in sentences (e.g. moving a limb in the same or opposite direction). In this task, higher reaction times usually result in the incompatible condition, but there are exceptions, and a debate concerning the very effect has developed in the literature. One subject of discussion is whether the assumed “internal simulation” involves a representation of space, or the preparation of some bodily motion. In order to clarify this point, we performed a series of experiments where the sensibleness of sentences was judged using a mouse, which involves a representation of spatial relations but does not require a full motion. Disentangling this question is important in order to understand the role of body in language comprehension, assumed by the perspective of “embodiment”. For example how knowledge of actions in a situation, or how linguistic structures cooperate with action-related knowledge in order to enhance understanding and learning.
